# Supplementary figures and images for: The spatial-temporal distribution of soil-transmitted helminth infections in Guangdong Province, China: A geostatistical analysis of data derived from the three national parasitic surveys
Source: PLoS Negl Trop Dis. 2022 Jul 18;16(7):e0010622. doi: 10.1371/journal.pntd.0010622 (PMC9333454; doi:10.1371/journal.pntd.0010622)

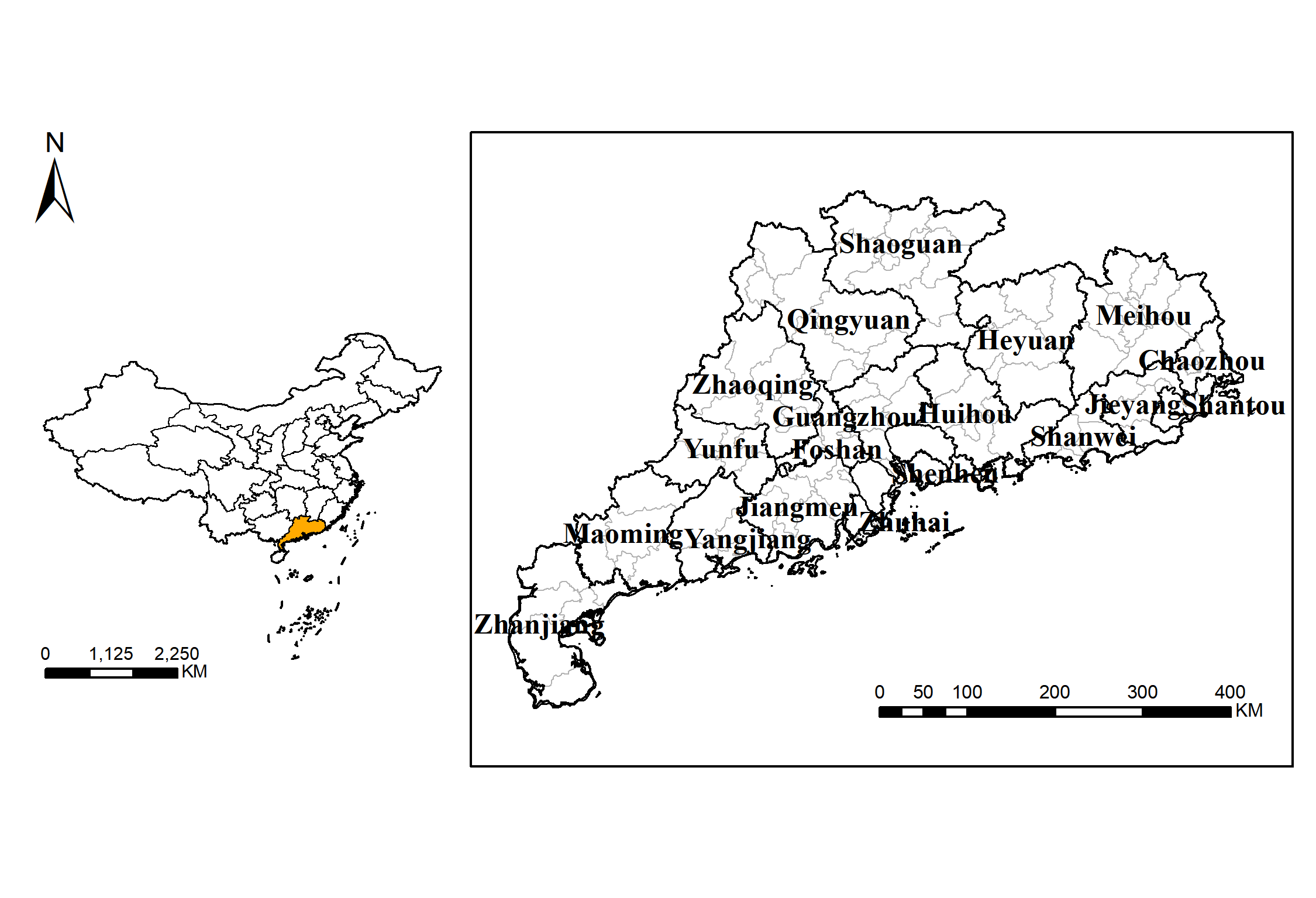

Supplement: S1 Fig — The base layer derived from https://www.webmap.cn/mapDataAction.do?method=forw&keysearch=indexSearch with credit to National Catalogue Service For Geographic Information. (TIF) [file pntd.0010622.s001.tif]

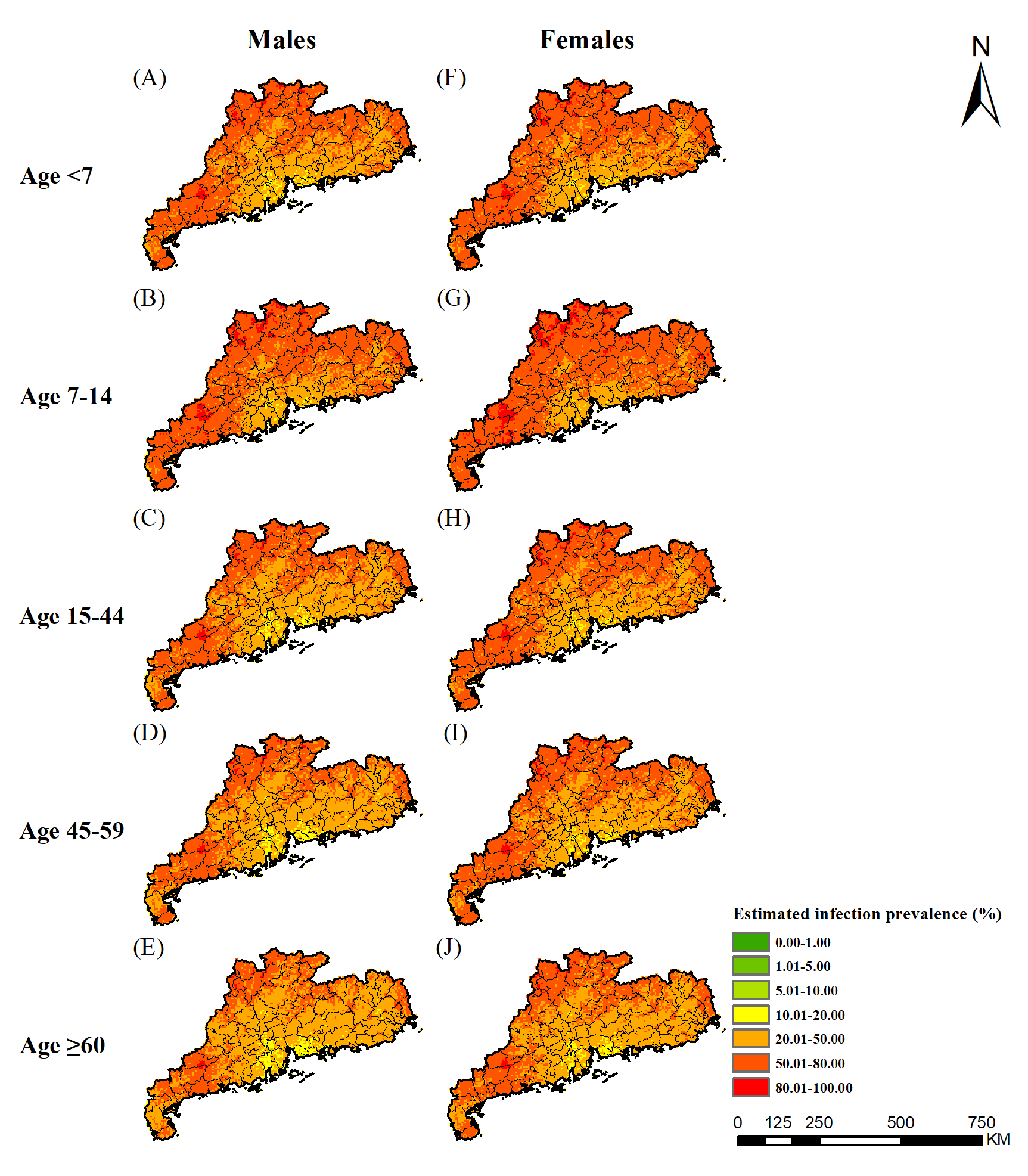

Supplement: S2 Fig — (A)-(E) present A. lumbricoide infection of males 0 to 6, 7 to 14, 15 to 44, 45 to 59, and 60 years old and older, (F)-(J) present A. lumbricoide infection of females 0 to 6, 7 to 14, 15 to 44, 45 to 59, and 60 years old and older, respectively. The base layer derived from https://www.webmap.cn/mapDataAction.do?method=forw&keysearch=indexSearch with credit to National Catalogue Service For Geographic Information. (TIF) [file pntd.0010622.s002.tif]

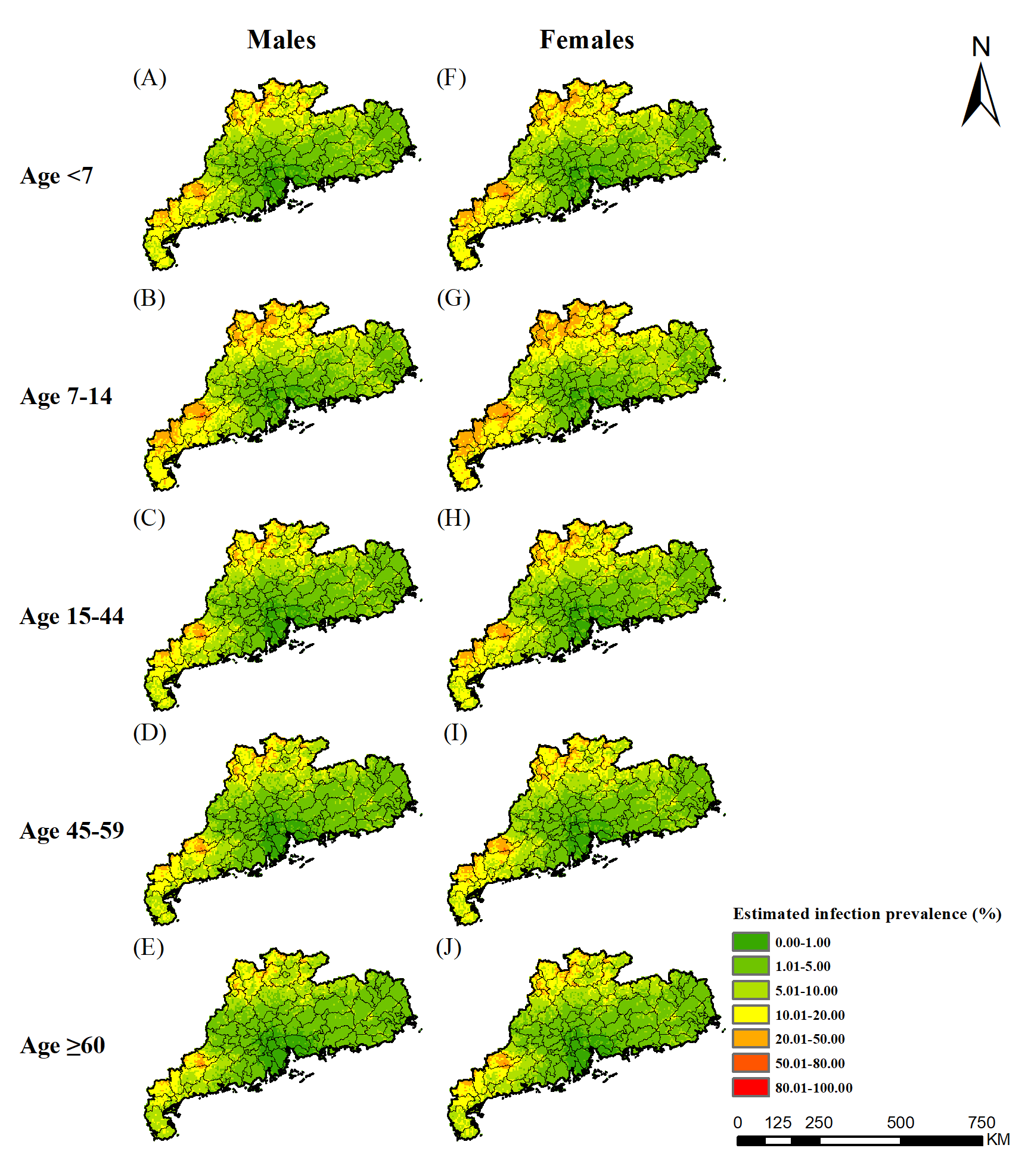

Supplement: S3 Fig — (A)-(E) present A. lumbricoide infection of males 0 to 6, 7 to 14, 15 to 44, 45 to 59, and 60 years old and older, (F)-(J) present A. lumbricoide infection of females 0 to 6, 7 to 14, 15 to 44, 45 to 59, and 60 years old and older, respectively. The base layer derived from https://www.webmap.cn/mapDataAction.do?method=forw&keysearch=indexSearch with credit to National Catalogue Service For Geographic Information. (TIF) [file pntd.0010622.s003.tif]

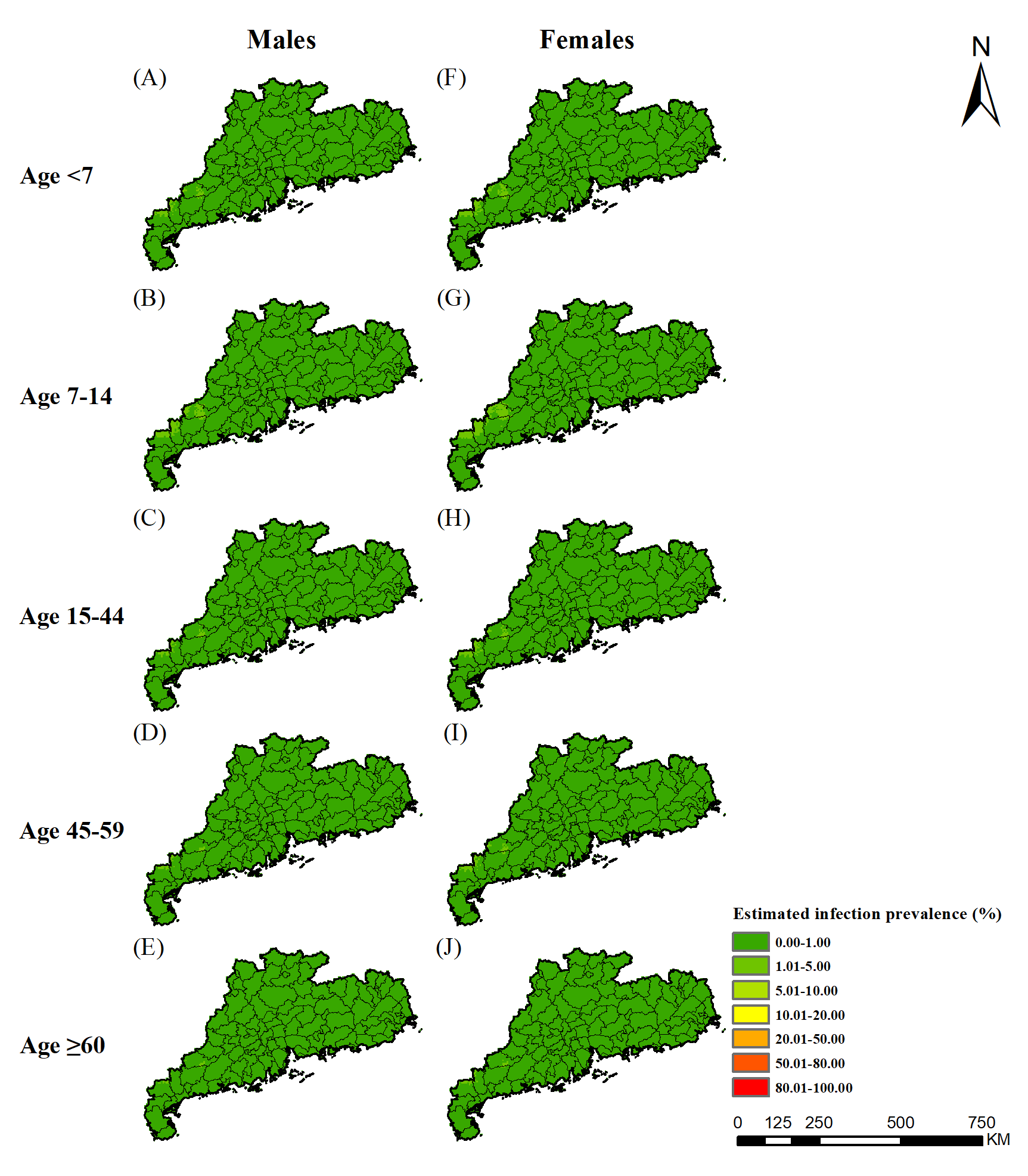

Supplement: S4 Fig — (A)-(E) present A. lumbricoide infection of males 0 to 6, 7 to 14, 15 to 44, 45 to 59, and 60 years old and older, (F)-(J) present A. lumbricoide infection of females 0 to 6, 7 to 14, 15 to 44, 45 to 59, and 60 years old and older, respectively. The base layer derived from https://www.webmap.cn/mapDataAction.do?method=forw&keysearch=indexSearch with credit to National Catalogue Service For Geographic Information. (TIF) [file pntd.0010622.s004.tif]

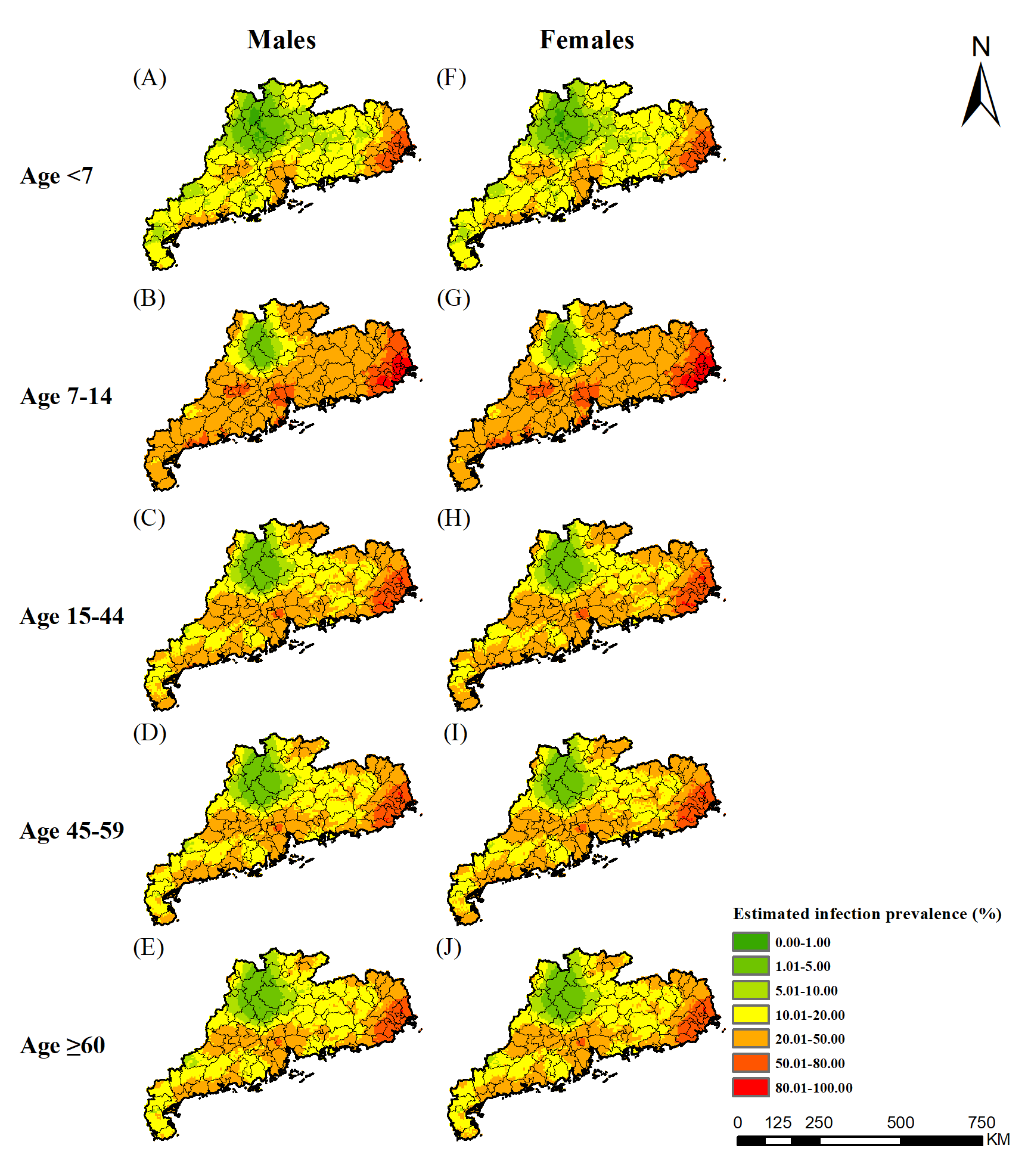

Supplement: S5 Fig — (A)-(E) present T. trichiura infection of males 0 to 6, 7 to 14, 15 to 44, 45 to 59, and 60 years old and older, (F)-(J) present T. trichiura infection of females 0 to 6, 7 to 14, 15 to 44, 45 to 59, and 60 years old and older, respectively. The base layer derived from https://www.webmap.cn/mapDataAction.do?method=forw&keysearch=indexSearch with credit to National Catalogue Service For Geographic Information. (TIF) [file pntd.0010622.s005.tif]

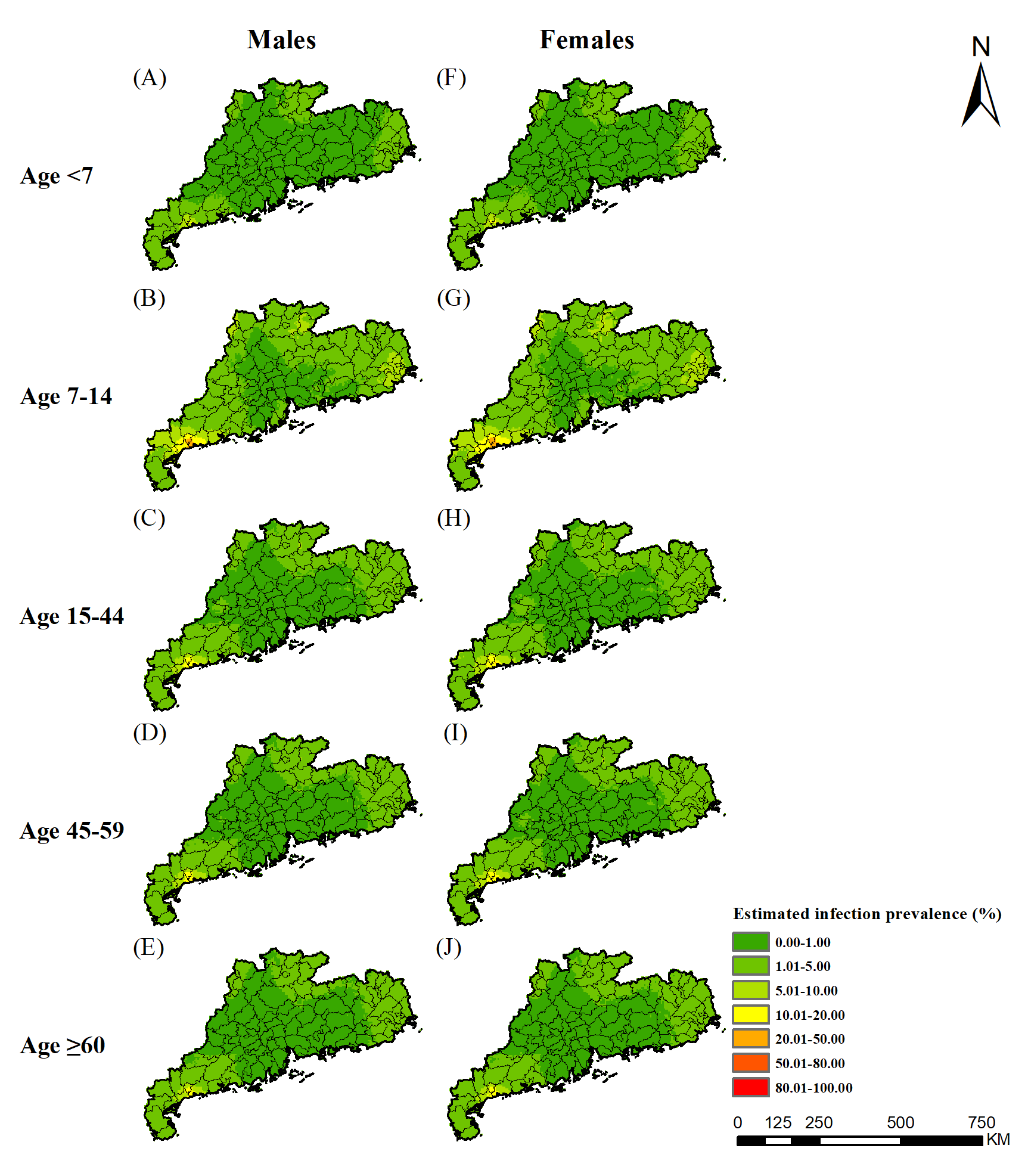

Supplement: S6 Fig — (A)-(E) present T. trichiura infection of males 0 to 6, 7 to 14, 15 to 44, 45 to 59, and 60 years old and older, (F)-(J) present T. trichiura infection of females 0 to 6, 7 to 14, 15 to 44, 45 to 59, and 60 years old and older, respectively. The base layer derived from https://www.webmap.cn/mapDataAction.do?method=forw&keysearch=indexSearch with credit to National Catalogue Service For Geographic Information. (TIF) [file pntd.0010622.s006.tif]

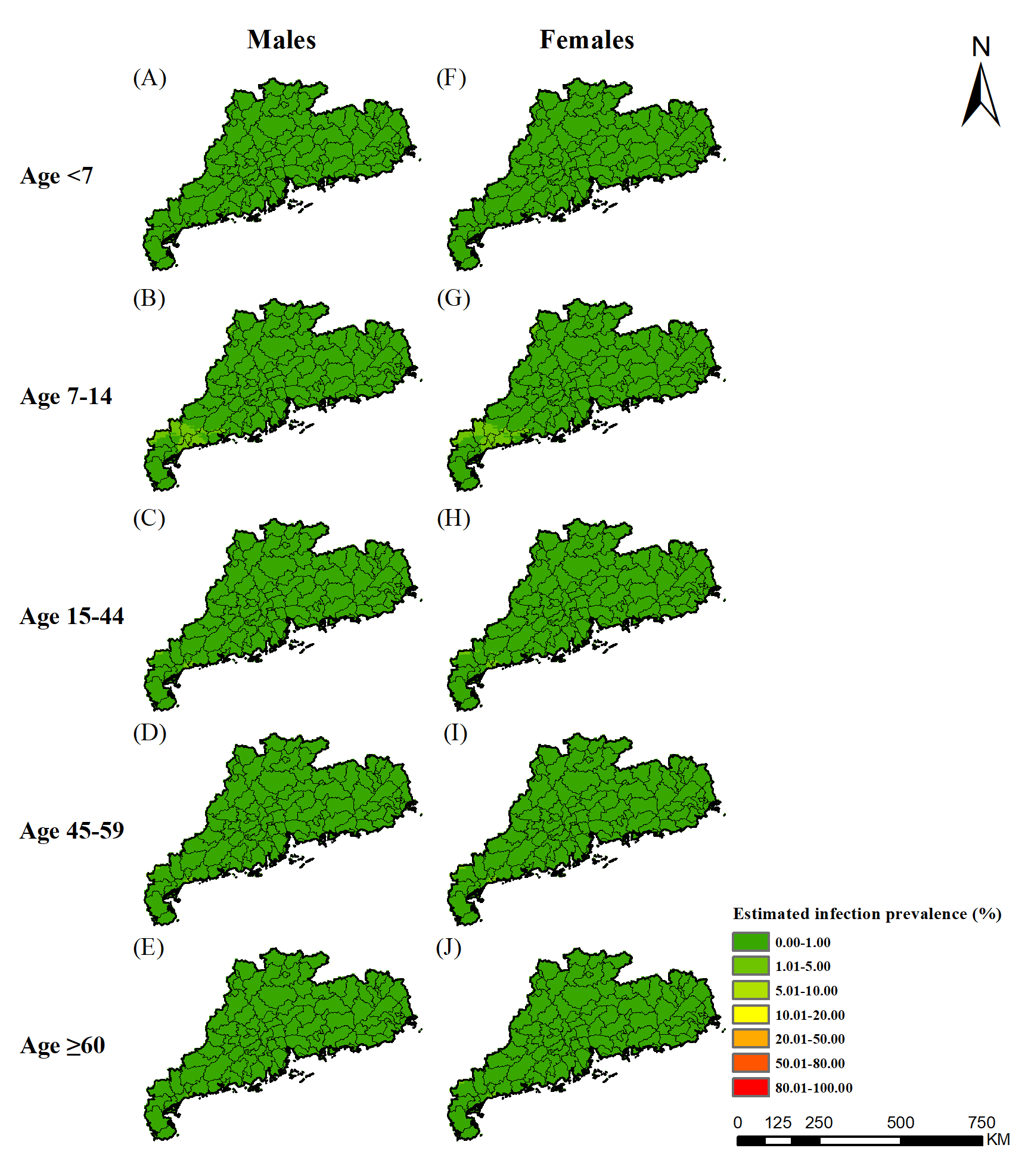

Supplement: S7 Fig — (A)-(E) present T. trichiura infection of males 0 to 6, 7 to 14, 15 to 44, 45 to 59, and 60 years old and older, (F)-(J) present T. trichiura infection of females 0 to 6, 7 to 14, 15 to 44, 45 to 59, and 60 years old and older, respectively. The base layer derived from https://www.webmap.cn/mapDataAction.do?method=forw&keysearch=indexSearch with credit to National Catalogue Service For Geographic Information. (TIF) [file pntd.0010622.s007.tif]

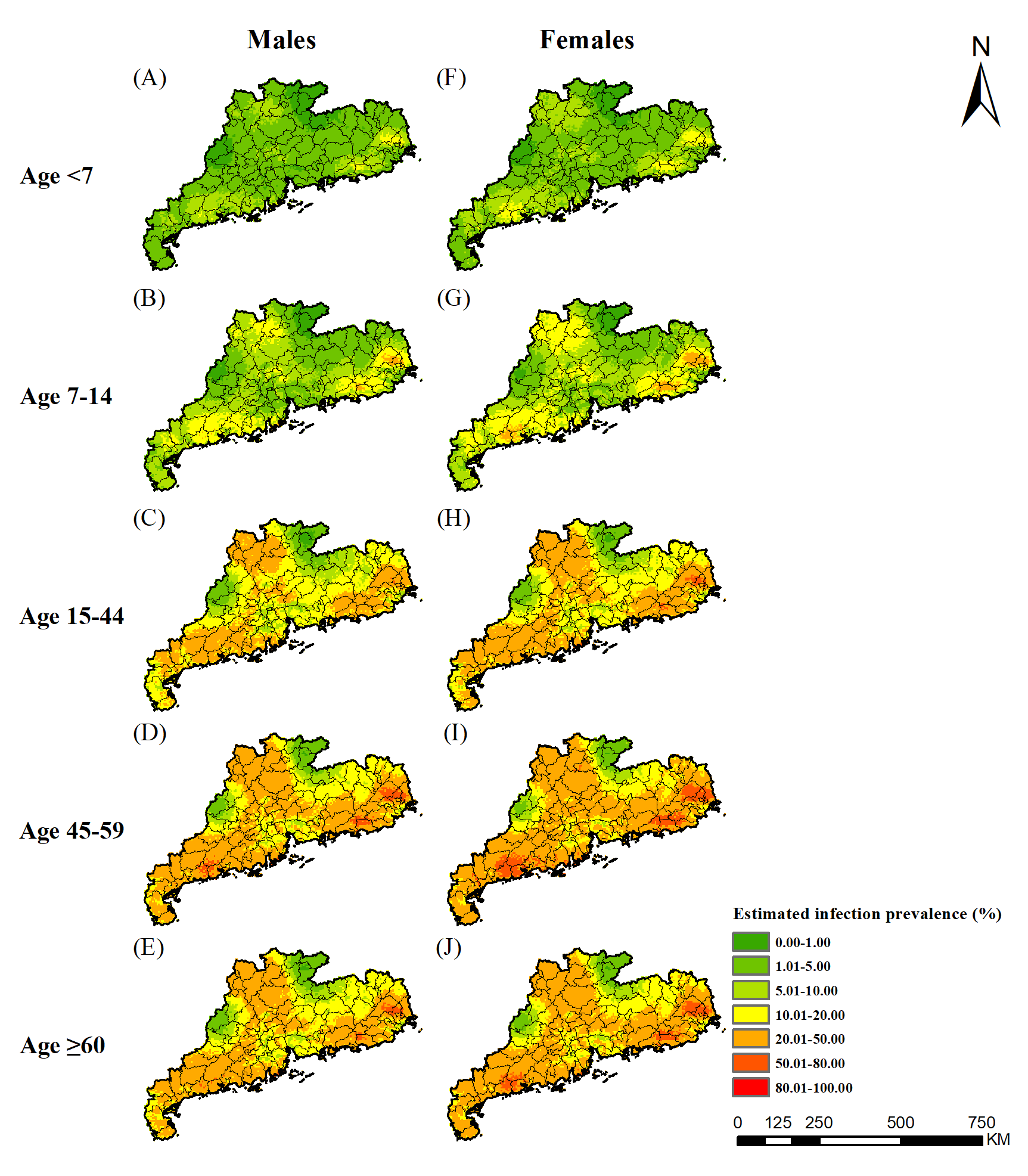

Supplement: S8 Fig — (A)-(E) present hookworm infection of males 0 to 6, 7 to 14, 15 to 44, 45 to 59, and 60 years old and older, (F)-(J) present hookworm infection of females 0 to 6, 7 to 14, 15 to 44, 45 to 59, and 60 years old and older, respectively. The base layer derived from https://www.webmap.cn/mapDataAction.do?method=forw&keysearch=indexSearch with credit to National Catalogue Service For Geographic Information. (TIF) [file pntd.0010622.s008.tif]

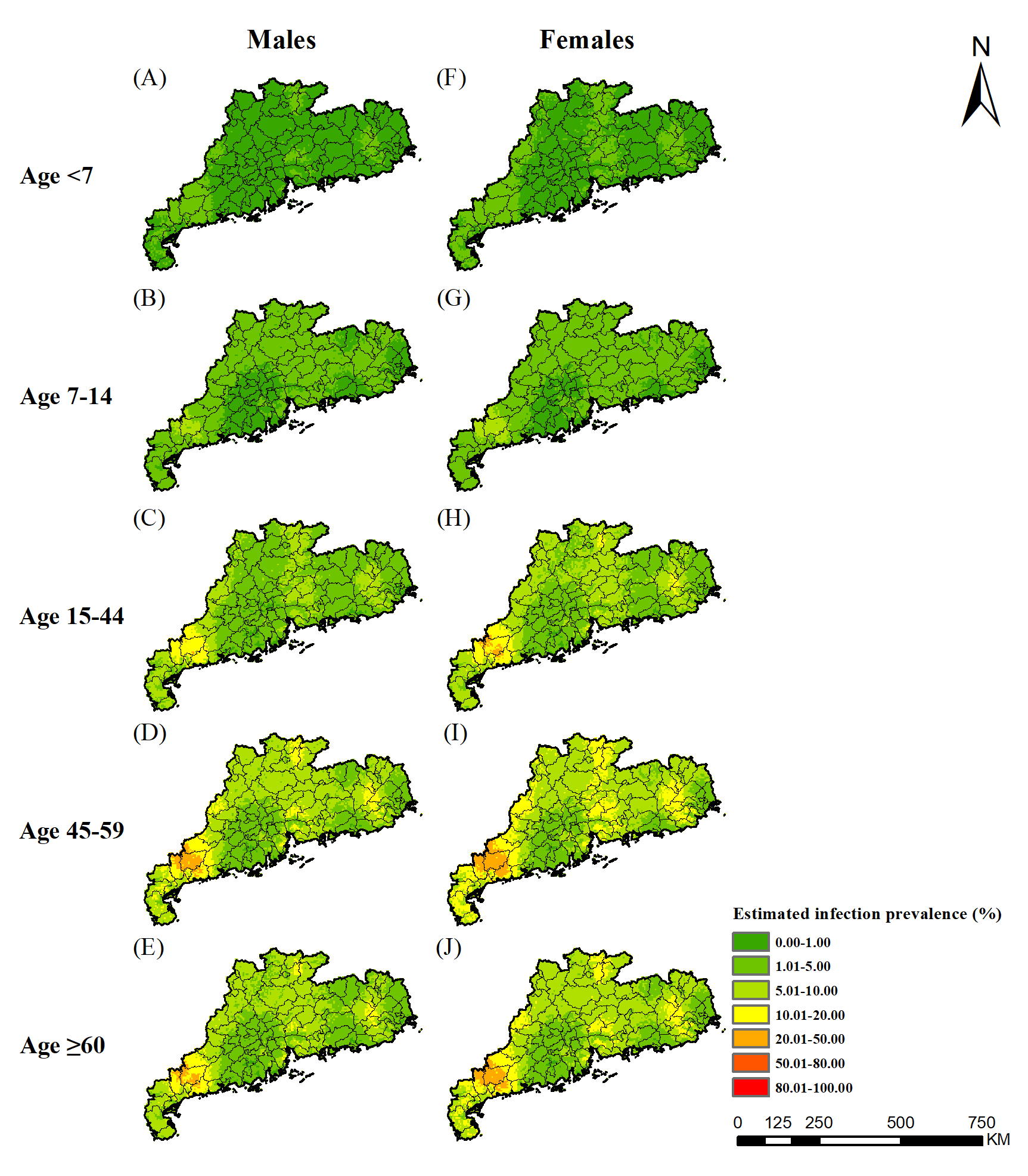

Supplement: S9 Fig — (A)-(E) present hookworm infection of males 0 to 6, 7 to 14, 15 to 44, 45 to 59, and 60 years old and older, (F)-(J) present hookworm infection of females 0 to 6, 7 to 14, 15 to 44, 45 to 59, and 60 years old and older, respectively. The base layer derived from https://www.webmap.cn/mapDataAction.do?method=forw&keysearch=indexSearch with credit to National Catalogue Service For Geographic Information. (TIF) [file pntd.0010622.s009.tif]

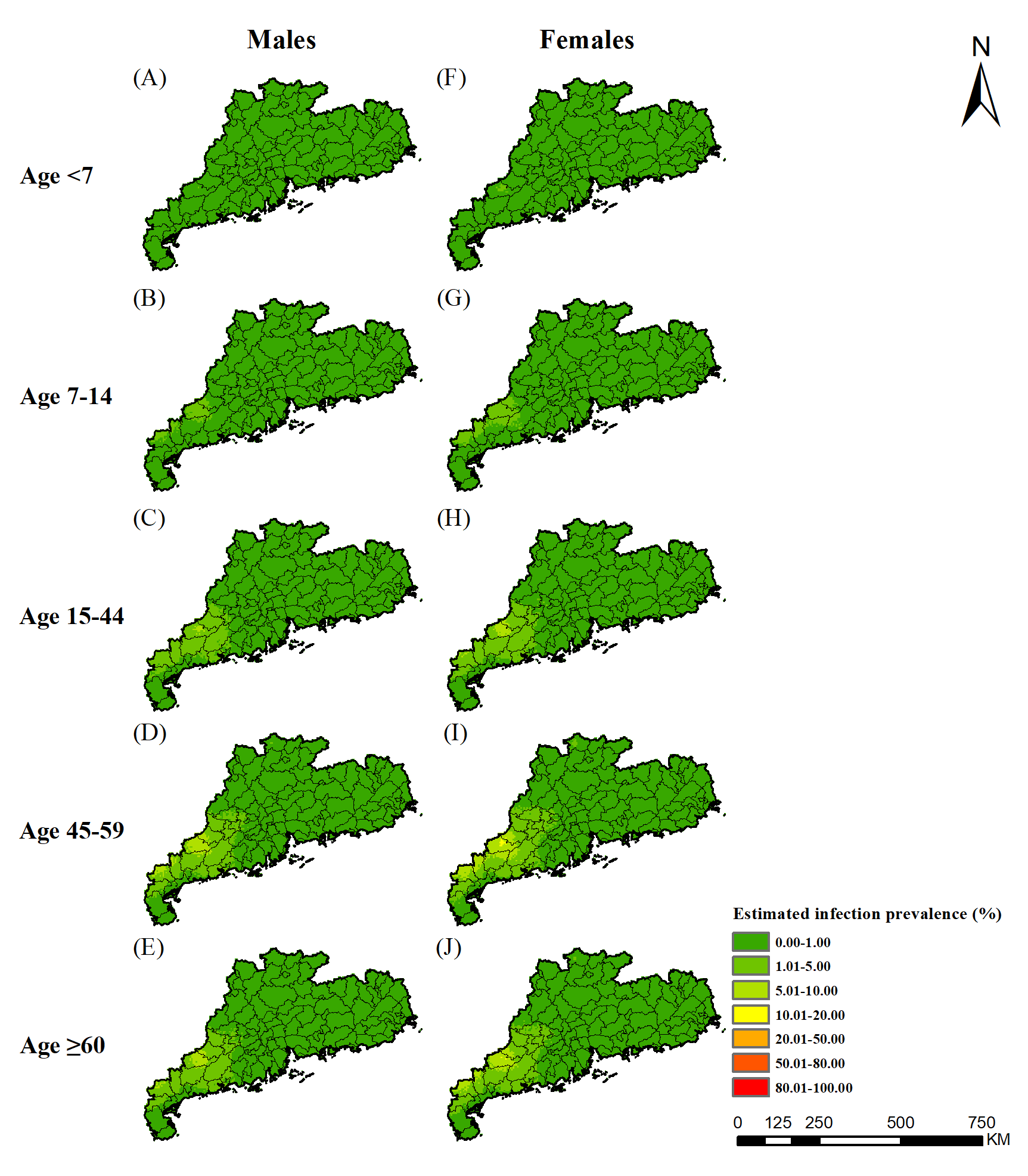

Supplement: S10 Fig — (A)-(E) present hookworm infection of males 0 to 6, 7 to 14, 15 to 44, 45 to 59, and 60 years old and older, (F)-(J) present hookworm infection of females 0 to 6, 7 to 14, 15 to 44, 45 to 59, and 60 years old and older, respectively. The base layer derived from https://www.webmap.cn/mapDataAction.do?method=forw&keysearch=indexSearch with credit to National Catalogue Service For Geographic Information. (TIF) [file pntd.0010622.s010.tif]
