# Supplementary material for: The spatial-temporal distribution of soil-transmitted helminth infections in Guangdong Province, China: A geostatistical analysis of data derived from the three national parasitic surveys
Source: PLoS Negl Trop Dis. 2022 Jul 18;16(7):e0010622. doi: 10.1371/journal.pntd.0010622 (PMC9333454; doi:10.1371/journal.pntd.0010622)
Supplement: S1 Text — (DOCX) [file pntd.0010622.s011.docx]

**S1 Text. The results of model validation**

**Table A. The results of model validation.**

| **Models of STH infections** | **ME** | **MAE** | **Within 95% BCI** | **AUC** |
| --- | --- | --- | --- | --- |
| *A. lumbricoides* | 0.88% | 9.43% | 82.3% | 0.95 |
| *T. trichiura* | -1.06% | 7.59% | 70.9% | 0.94 |
| Hookworm | 4.74% | 8.18% | 71.8% | 0.88 |
| Any STH | 0.22% | 10.89% | 81.0% | 0.95 |


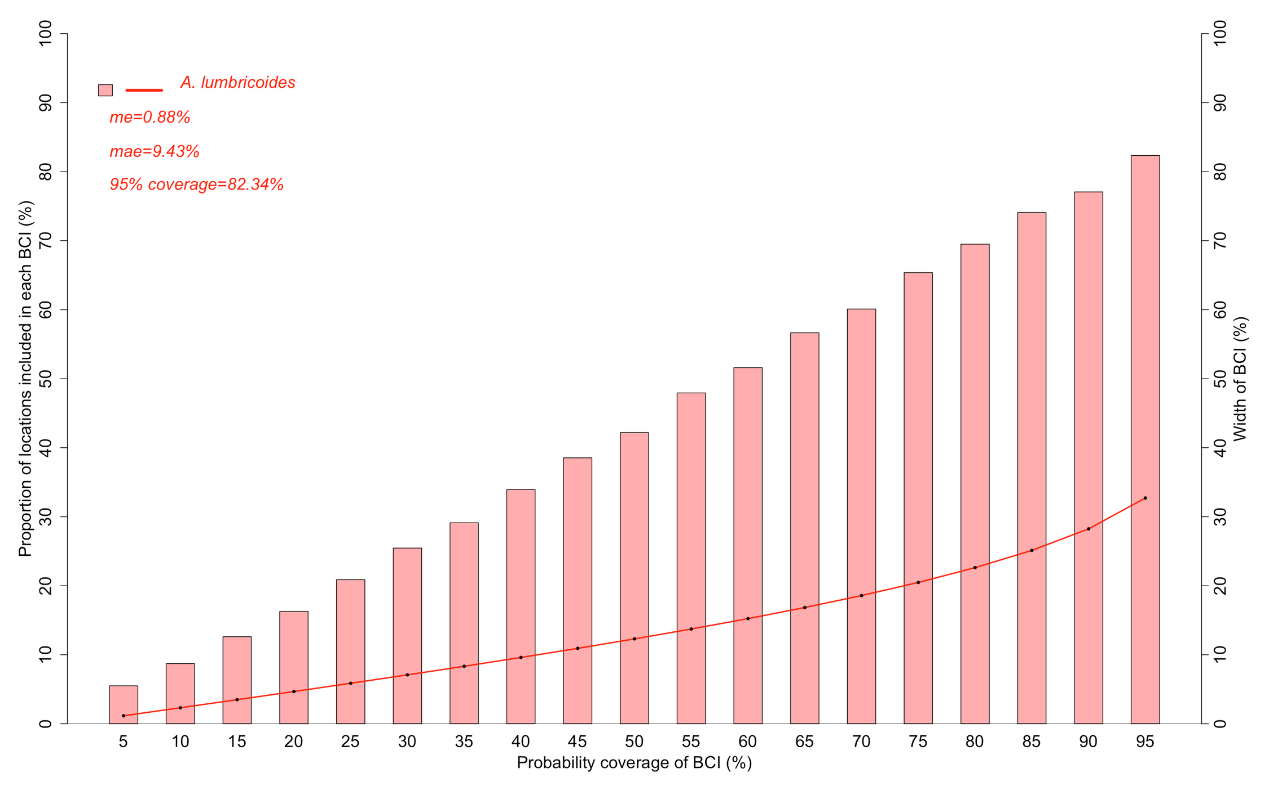


**Fig A. The ME, MAE, and coverage of locations within 95% BCI of model validation for *A. lumbricoides* infection.**





**Fig B. The AUC of ROC of model validation for *A. lumbricoides* infection.**


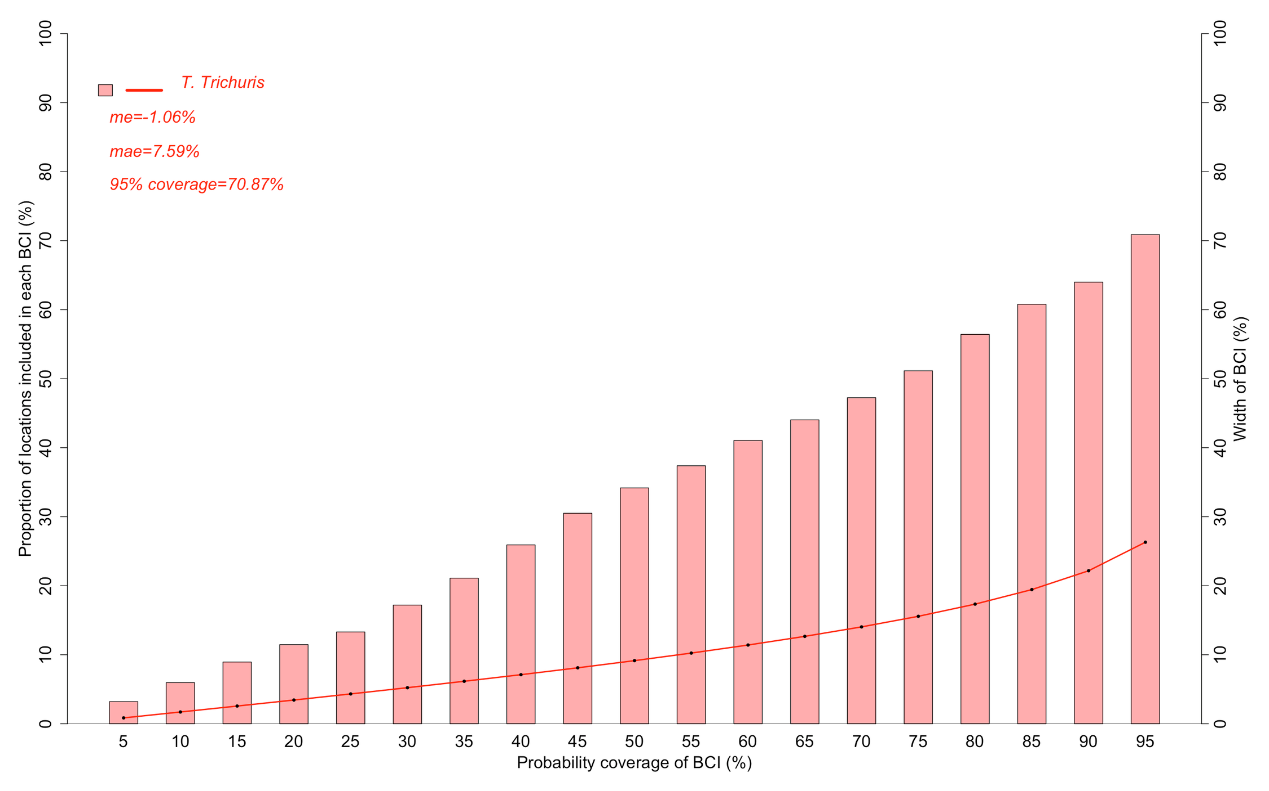


**Fig C. The ME, MAE, and coverage of locations within 95% BCI of model validation for *T. trichiura* infection.**





**Fig D. The AUC of ROC of model validation for *T. trichiura* infection.**


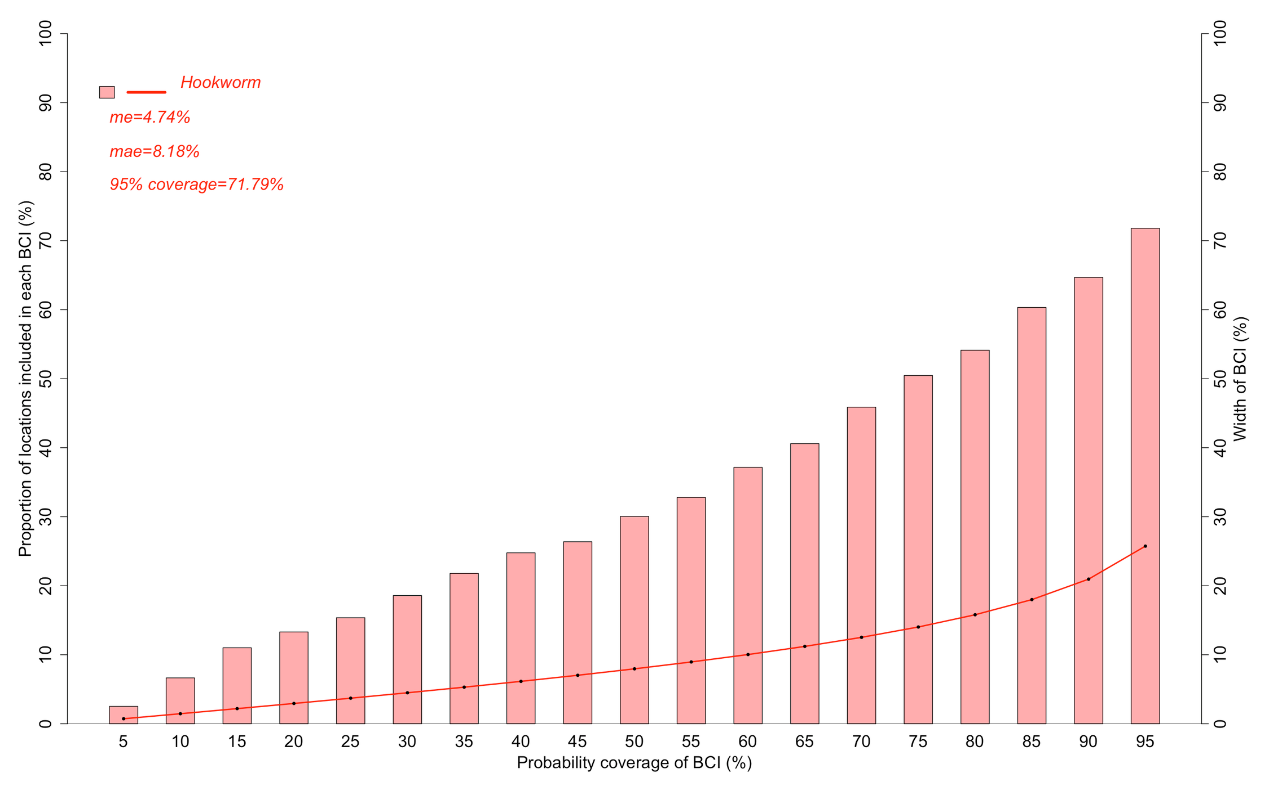


**Fig E. The ME, MAE, and coverage of locations within 95% BCI of model validation for hookworm infection.**





**Fig F. The AUC of ROC of model validation for hookworm infection.**


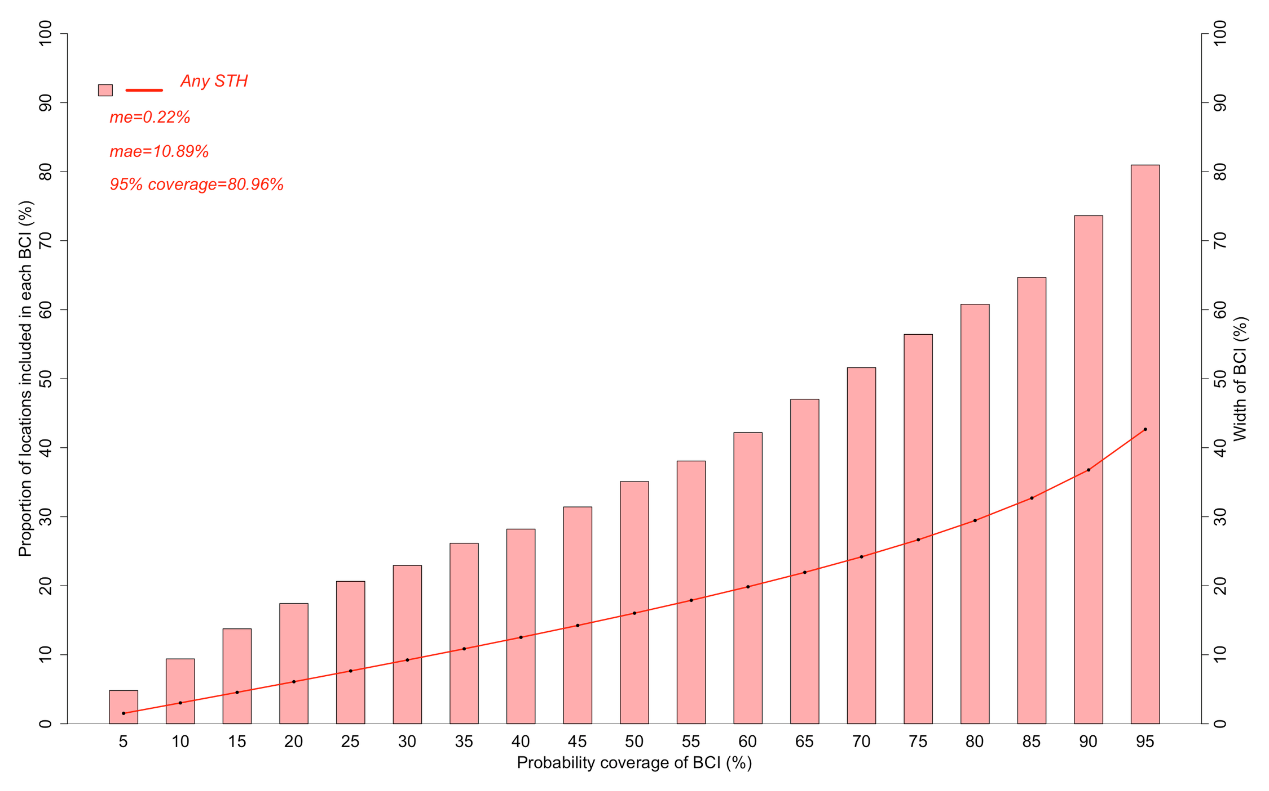


**Fig G. The ME, MAE, and coverage of locations within 95% BCI of model validation for any STH infection.**





**Fig H. The AUC of ROC of model validation for any STH infection.**
