# Supplementary material for: The spatial-temporal distribution of soil-transmitted helminth infections in Guangdong Province, China: A geostatistical analysis of data derived from the three national parasitic surveys
Source: PLoS Negl Trop Dis. 2022 Jul 18;16(7):e0010622. doi: 10.1371/journal.pntd.0010622 (PMC9333454; doi:10.1371/journal.pntd.0010622)
Supplement: Attachment — Submitted filename: STH spatial Kenya.pdf [file pntd.0010622.s012.pdf]

PLoS NEGLECTED TROPICAL DISEASES

Spatial Modelling of Soil-Transmitted Helminth Infections in Kenya: A Disease Control Planning Tool

Rachel L. Pullan[1]*, Peter W. Gething[2], Jennifer L. Smith[1], Charles S. Mwandawiro[3], Hugh J. W. Sturrock[1], Caroline W. Gitonga[4], Simon I. Hay[2], Simon Brooker[1,4]

1 Department of Infectious and Tropical Diseases, London School of Hygiene and Tropical Medicine, London, United Kingdom, **2** Spatial Ecology and Epidemiology Group, Department of Zoology, University of Oxford, Oxford, United Kingdom, **3** Kenya Medical Research Institute (KEMRI), Nairobi, Kenya, **4** KEMRI-Wellcome Trust Research Programme, Nairobi, Kenya

Abstract

Background: Implementation of control of parasitic diseases requires accurate, contemporary maps that provide intervention recommendations at policy-relevant spatial scales. To guide control of soil transmitted helminths (STHs), maps are required of the combined prevalence of infection, indicating where this prevalence exceeds an intervention threshold of 20%. Here we present a new approach for mapping the observed prevalence of STHs, using the example of Kenya in 2009.

Methods and Findings: Observed prevalence data for hookworm, *Ascaris lumbricoides* and *Trichuris trichiura* were assembled for 106,370 individuals from 945 cross-sectional surveys undertaken between 1974 and 2009. Ecological and climatic covariates were extracted from high-resolution satellite data and matched to survey locations. Bayesian space-time geostatistical models were developed for each species, and were used to interpolate the probability that infection prevalence exceeded the 20% threshold across the country for both 1989 and 2009. Maps for each species were integrated to estimate combined STH prevalence using the law of total probability and incorporating a correction factor to adjust for associations between species. Population census data were combined with risk models and projected to estimate the population at risk and requiring treatment in 2009. In most areas for 2009, there was high certainty that endemicity was below the 20% threshold, with areas of endemicity ≥20% located around the shores of Lake Victoria and on the coast. Comparison of the predicted distributions for 1989 and 2009 show how observed STH prevalence has gradually decreased over time. The model estimated that a total of 2.8 million school-age children live in districts which warrant mass treatment.

Conclusions: Bayesian space-time geostatistical models can be used to reliably estimate the combined observed prevalence of STH and suggest that a quarter of Kenya's school-aged children live in areas of high prevalence and warrant mass treatment. As control is successful in reducing infection levels, updated models can be used to refine decision making in helminth control.

Citation: Pullan RL, Gething PW, Smith JL, Mwandawiro CS, Sturrock HJW, et al. (2011) Spatial Modelling of Soil-Transmitted Helminth Infections in Kenya: A Disease Control Planning Tool. PLoS Negl Trop Dis 5(2): e958. doi:10.1371/journal.pntd.0000958

Editor: Giovanna Raso, Centre Suisse de Recherches Scientifiques, Côte d'Ivoire

Received July 28, 2010; **Accepted** January 7, 2011; **Published** February 8, 2011

Copyright: © 2011 Pullan et al. This is an open-access article distributed under the terms of the Creative Commons Attribution License, which permits unrestricted use, distribution, and reproduction in any medium, provided the original author and source are credited.

Funding: RLP is supported by the Partnership for Child Development, Imperial College, UK, who, together with Deworm the World, principally fund the Global Atlas of Helminth Infection (GAHI, www.thiswormyworld.org). SB is supported by a Research Career Development Fellowship (#081673) from the Wellcome Trust, which also supports the GAHI. JLS is supported by the International Trachoma Initiative to develop a Global Atlas of Trachoma. SIH is funded by a Senior Research Fellowship from the Wellcome Trust (#079091), which also supports PWG. HS is supported by a London School of Hygiene and Tropical Medicine Graduate Teaching Assistantship. This work forms part of the output of the Malaria Atlas Project (MAP, www.map.ox.ac.uk), principally funded by the Wellcome Trust, UK. The funders had no role in the study design, data collection and analysis or preparation of the manuscript.

Competing Interests: The authors have declared that no competing interests exist.

* E-mail: rachel.pullan@lshtm.ac.uk

Introduction

In Africa, an increasing number of countries are implementing national treatment programmes for the control of soil-transmitted helminths (STH). The main strategy of these programmes is the delivery of deworming through the public school system, which has been demonstrated as a cost-effective way to reduce infection and morbidity of STH and improve educational outcomes [1–4]. There have also been moves to integrate mass drug administration (MDA) for STH and schistosomiasis with other neglected tropical diseases (NTDs), including lymphatic filariasis (LF) and onchocerciasis [5,6]. Whatever the implementation approach, governments need to target treatment appropriately, based on reliable and up-to-date information on the geographical distribution of infection [7]. The ability to map the distribution of STHs has been greatly enhanced in recent years by the use of geographical information systems, remote sensing and spatial statistics [8,9]. Recent work has increasingly employed a Bayesian geostatistical modelling approach, which provides a flexible and powerful tool for risk mapping, enabling investigation of spatial heterogeneity, environmental predictors and associated uncertainties. To date, Bayesian geostatistics has been used to predict spatial patterns for a number of NTDs, including STH [10–12], schistosomiasis [13–15], *Loa loa* [16] and LF [17], facilitating prediction of infection prevalence in unsurveyed areas, targeting of large-scale control programmes and prioritisation of future data collection. Whilst the derived risk maps

Author Summary

Effective targeting of mass drug administration for the treatment of soil-transmitted helminths (STH) requires reliable, up-to-date maps that indicate where prevalence exceeds the 20% intervention threshold recommended by the World Health Organization. We present a new approach for mapping the prevalence of STH in Kenya, incorporating observed prevalence data from 945 cross-sectional surveys undertaken between 1974 and 2009. The distribution of each species was modelled using model-based geostatistics; models included information on environmental factors, the spatial distribution of existing surveys and when these surveys were conducted. Resulting risk maps were combined and linked with population data enabling estimation of the population at risk of any STH infection and requiring treatment in 2009. In most areas, there was high certainty that combined STH prevalence was below the 20% intervention threshold, with areas of high prevalence located around the shores of Lake Victoria and on the coast. Results also suggest that observed prevalence decreased over time and emphasise the importance of continued surveillance in areas where observed prevalence was historically high. We show how spatial modelling can be used to develop up-to-date maps of STH risk to help improve the precision of decision making in disease control.

are statistically appealing, their uptake by managers of national control programmes could be improved. This is in part due to difficulty in interpreting these continuous, species-specific maps and translating them into practical guidance for STH control at district levels, which is defined on the basis of combined prevalence of any STH species. Furthermore, the extent to which models can be extrapolated temporally remains unclear [13,18]. This is particularly relevant when decision makers need to rely on historic data to implement control in a rapidly changing epidemiological context, due to both general improvements in socio-economic and sanitary conditions and previous deworming efforts.

In this study, we use the largest assembly of contemporary empirical evidence for STH in any country in Africa to map the observed prevalence of STH across Kenya using a space-time model based geostatistical approach, and estimate the spatially-varying probability that mass treatment is warranted. The resulting models are used to calculate the school-aged population at risk of infection and requiring MDA in Kenya.

Methods

Analysis outline

The objective of these analyses was to determine the spatial distribution of observed prevalence of STH infection in Kenya. Data on the observed prevalence of hookworm, *Ascaris lumbricoides* and *Trichuris trichiura* spanning 34 years were collated using search principles and criteria outlined below, in order to create a robustly geo-located dataset of helminth surveys. This database was used to produce a continuous predictive surface of combined observed STH prevalence adopting a Bayesian space-time geostatistics approach, adjusting for environmental covariates; no spatial prediction was made for areas masked as environmentally unsuitable for STH transmission (maximum land surface temperature (LST) >40°C). The resulting models were used to interpolate the probability across Kenya that combined observed STH prevalence is greater than 20%, the threshold recommended by the World Health Organization (WHO) as indicating the need for targeted MDA [19]. Validation procedures were implemented to assess the accuracy of endemicity predictions. Finally, the total population at risk of STH infection was extracted by district in order to guide targeted intervention strategies.

Data sources

Data on the prevalence of infection in Kenya were abstracted from an ongoing project to develop a *Global Atlas of Helminth Infection* (www.thiswormyworld.org; [7]). For this project, survey data are identified through structured searches of electronic bibliographic databases, complemented with manual searches of local archives and libraries and direct contact with researchers. A manual search of archives of the Division of Vector Borne and Neglected Tropical Diseases of the Kenya Ministry of Public Health and Sanitation in Nairobi proved a particularly useful source of information. References from identified publications were checked for additional surveys. Estimates of infection prevalence were included according to pre-defined criteria: only cross-sectional prevalence surveys were included; data were excluded if based on hospital or clinic surveys, post-intervention surveys, or surveys among sub-populations, such as among refugees, prisoners or nomads. For data from clinical trials or cohort studies, only baseline, pre-intervention estimates of prevalence were included. In instances where multiple surveys from the same location were conducted at different times, each survey was included. Abstracted data included details on the source of the data, date and location of survey, characteristics of the surveyed population, survey methodology, method of diagnosis, age range of sampled individuals, and the number of individuals examined and the number positive with hookworm, *A. lumbricoides* and *T. trichiura*. Authors of published data were contacted if relevant information was unclear from the original reports. For the current analysis, survey data were collected between 1974 and 2009.

The longitude and latitude of each survey were determined using a combination of resources including a national schools database developed by the Ministry of Education in 2008 using geographical positioning systems (GPS); a village database digitised from topographical maps in 2002 by the International Livestock Research Institute in Nairobi; a range of electronic gazetteers (see Brooker et al. [7]); and contact with authors who used GPS.

Estimating combined prevalence of any STH

Survey reports typically provide proportions infected with individual worm species, and rarely give data on the combined proportion infected with any STH species, although it is the prevalence of infection with any STH species (i.e. combined prevalence of all STH) that is important when making decisions about effective targeting for treatment. Therefore, combined STH prevalence was calculated using a simple probabilistic model, incorporating a small correction factor to allow for non-independence between species, following the approach of de Silva and Hall [20]. In brief, when assuming the probability of infection with one species to be independent of infection with others, the combined probability of having at least one infection is the simple probability law for the union between three elements: $P_{HAT} = H + A + T - (HA) - (AT) - (HT) + (HAT)$ where P_{HAT} is the combined STH prevalence, H is the prevalence of hookworm infection, A the prevalence of *A. lumbricoides* and T the prevalence of *T. trichiura*. Previous analysis of 60 datasets from 20 countries by de Silva and Hall suggests that, due to non-independence, overestimation of combined STH prevalence using simple probability increases by 0.6% for every 10% increase in

prevalence [20]. The true combined observed prevalence of STH can therefore be estimated as $P_{HAT} \div 1.06$. This correction factor was thus incorporated into the estimation of combined observed prevalence of STH. This approach to estimating combined prevalence is used, both when presenting observed prevalence data and when using species specific models to develop spatial models of combined observed prevalence, as explained below.

Ecological and climatic covariates and limits of transmission

A set of ecological and climatic covariates were assembled from a variety of sources, as described elsewhere [21]. Monthly average LST and precipitation at 30-arcsec (~1 km) resolution were downloaded from the WorldClim website [22]. These were produced from global weather station temperature records gathered from a variety of sources for the period 1950–2000 and interpolated using a thin-plate smoothing spline algorithm [23]. Enhanced vegetation index (EVI; a measure of vegetation density) for 2001–2005 were obtained from the Moderate Resolution Imaging Spectroradiometer (MODIS) [24], and elevation was obtained from an interpolated digital elevation model from the Global Land Information System of the United States Geological Survey ((http://edcwww.cr.usgs.gov/landdaac/gtopo30/). Distance to permanent water bodies was derived in ArcMap 9.2 from an electronic map obtained from the World Wildlife Fund (Global 200 Ecoregions data [25]). Maximum LST, elevation and precipitation were standardised to optimise sampling during Markov Chain Monte Carlo (MCMC) simulation by subtracting the arithmetic mean and dividing by the standard deviation.

These ecological and climatic data, along with results from previous studies, were used to define the spatial limits for the transmission of STH. Specifically, it has been shown experimentally that the development of free-living infectious stages of *A. lumbricoides* and *T. trichiura* ceases at 38°C and hookworm at 40°C [26–29]. This is supported by an observed relationship between prevalence across sub-Saharan Africa and maximum LST, shown in Figure 1. On this basis, using monthly LST at 30–arcsec (~1 km) spatial resolution [22], areas were masked as unsuitable for STH transmission where maximum LST >40°C. No spatial prediction was subsequently made for such areas.

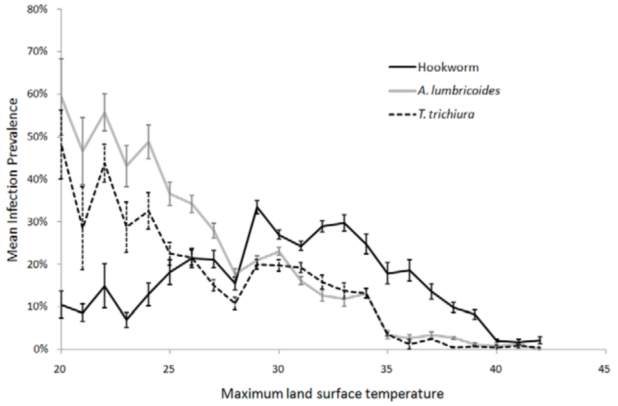

Figure 1. Relationship between mean land surface temperature (LST) and prevalence of soil-transmitted helminth infection. LST estimated from global weather station temperature records, Estimates are derived from 3,714 spatially unique cross-sectional survey locations across sub-Saharan Africa, provided by the Global Atlas of Helminth Infection (www.thiswormyworld.org). Error bars show the standard error of the mean.
doi:10.1371/journal.pntd.0000958.g001

Bayesian space-time modelling approach

Prevalence of hookworm, *A. lumbricoides* and *T. trichiura* were modelled separately. Variable selection from abstracted survey covariates (age range of sampled individuals, method of diagnosis) and environmental covariates was performed using fixed-effects grouped logistic regression models in Stata/SE 10.0 (StataCorp, College Station, TX, USA) with backwards elimination. Bayesian space time multivariable models were subsequently developed in WinBUGs version 1.4 (MRC Biostatistics Unit, Cambridge and Imperial College London, UK) (see **Text S1**). The number of examined and egg-positive individuals for each species at each survey location were modelled as binomial variates (i.e. grouped hierarchical logistic regression), conditional on a vector of fixed environmental covariate effects as described above and a geostatistical random effect that modelled spatial correlation using an isotropic, stationary exponential decay function [30]. To capture changes in prevalence over time, a temporal first-order autoregressive function was also included, under the assumption that fitted temporal correlation exists only with the preceding year [18,31].

Spatial prediction

Predictions of infection prevalence were made on a 0.1×0.1 decimal degree (approx 11×11 km at the equator) grid covering Kenya, at every point considered suitable for STH transmission (i.e. maximum LST <40°C). Prediction was performed in WinBUGs using the *spatial.unipred* command, which implements an interpolation function (krigging) for the spatial random effect. Kato-Katz is the WHO recommended diagnostic for surveillance of STH infections [32,33], and mass drug administration is targeted at school-aged children [16]. Thus, models were coded so that the predictive surfaces represented school-based surveys using the Kato-Katz technique for diagnosis. Predicted prevalence at each realisation was calculated for two years (1989, 2009), chosen to provide both a historical and contemporary representation of infection levels. Prediction was carried out by adding the interpolated geostatistical random effect and the temporal random effect corresponding with the year of interest to the sum of the products of the covariate coefficients and the values of the covariates at each prediction location. The overall sum was then back transformed from the logit scale to the prevalence scale. Prevalence of STH at each realisation was estimated using the probabilistic model given above, producing a posterior probability distribution for combined STH at each prediction location.

Probability contour maps were subsequently developed by calculating the observed proportion of the STH posterior probability distribution at each prediction location that exceeded WHO policy intervention thresholds (20% and 50% prevalence) [16]. Prediction locations were classified as endemic if the probability that observed STH prevalence exceeded 20% (the MDA once-yearly intervention threshold) was >0.5 (i.e. this was the most likely category). These locations were further classified as hyper-endemic if the probability that observed STH prevalence exceeded 50% (the MDA twice-yearly intervention threshold) was >0.5. Digital administration level 2 (district) boundaries and a population distribution map of Kenya (adjusted population counts for the year 2000 projected to 2009 by applying the national, medium variant, inter-censal growth rates, as previously described [34], at 5 km^2 resolution) were overlaid on the endemicity-class surface to determine the proportion of the district population living in each endemicity class. Districts were defined as MDA

intervention districts if over 33% of the population were in endemic (once-yearly MDA) or hyperendemic (twice yearly MDA) endemicity classes. This cut-off was chosen post-estimation as the level that ensures treatment of >80% of the population predicted as exceeding the 20% prevalence threshold. The proportion of the Kenyan population of school-going age (5–14 years) was estimated as 0.26, based on the World Population Prospects: 2008 Revision Population Database (http://esa.un.org/unpp/index.asp?panel = 3; accessed 11th May 2010) and the number of treatments needed estimated using a primary-school net enrolment rate of 0.815 (http://data.worldbank.org/indicator/SE.PRM.NENR; accessed 11th May 2010; [35]).

Model validation

Model validation was performed by randomly allocating the survey locations to one of four subsets (each containing approximately 25% of the total dataset) and training the space time models on the three subsets whilst simultaneously predicting infection prevalence at the locations of the fourth, excluded subset (as done previously [10]). This was repeated for each subset, giving an observed and predicted value for all 945 survey locations. Validation statistics included area under the curve (AUC) of the receiver operating characteristic (ROC; a plot of sensitivity vs. one minus specificity, where the predicted probability of infection being greater than 5%, 20% and 50% were compared to observed values) to assess discriminatory performance of the predictive model [36] AUC values of <0.7 indicate poor discriminatory performance, 0.7–0.8 acceptable, 0.8–0.9 excellent and >0.9 outstanding discriminatory performance. The correlation coefficient between observed and predicted prevalence and the mean error and mean absolute error were used to assess bias (mean error) and the accuracy (mean absolute error) of predictions. Validation was repeated for models without temporal and/or spatial random effects.

Results

Survey data

A total of 976 independent surveys conducted between 1975 and 2009 were identified through our searches, of which 945 surveys (97%), including some 106,370 individuals, could be geo-positioned. The majority (96%) of surveys were conducted among children in schools. Of the included surveys, 813 were spatially unique locations, whilst the rest were surveys undertaken in the same location but at different times.

Figure 2 presents the observed geographical distribution of each species as well as the estimated combined prevalence of STH, based on all the included survey data. The observed prevalence of all STH species is highest in western Kenya and on the coast; pockets of observed high prevalence of *A. lumbricoides* and *T. trichiura* prevalence also occur in central Kenya. In addition to geographical variation, observed prevalence also varies over time (**Table 1**): for example, mean combined STH prevalence in Central Province was 43.8% in the period 1980–1989 (n = 86 surveys), but only 12.6% in the period 2000–2009 (n = 80 surveys).

Bayesian space time modelling

In univariate analysis, all tested environmental covariates were statistically significant predictors of infection prevalence (See **Text S2**). In particular, infection prevalence was significantly associated with the diagnosis method used for all species. Prevalence was also significantly associated with maximum LST, elevation, precipitation, EVI and distance to permanent water bodies. Note that the odds ratios are on the same scale (standard deviations) for each variable, which were standardised to have a mean of zero and a standard deviation of one. Model-based geostatistical analysis resulted in some weakening of associations between environmental predictors and odds of infection, because these environmental covariates are themselves spatially correlated. Multivariable analysis (**Table 2**) indicated that the odds of *A. lumbricoides* was significantly lower in community-based surveys compared with school surveys and showed borderline associations with maximum LST. Estimates of *T. trichiura* odds were lower in community based surveys and in surveys that used alternative diagnostic methods to Kato Katz, significantly decreased with increasing maximum LST and elevation, and demonstrated a borderline negative association with precipitation. Odds of hookworm infection also differed significantly with diagnostic method, was significantly negatively associated with maximum LST and elevation, and demonstrated borderline positive associations with precipitation.

Variance of the geostatistical random effect (which indicates a propensity for clustering not explained by the environmental covariates) was similarly large for all three STH species. For all three species the distance at which spatial correlation fell below 5% was 39–49 km (95% credible intervals ranging between 31–68 km), corresponding with a very rapid decline in spatial correlation with distance at larger scales, after accounting for covariates. The variance of the temporal random effect was greatest for *A. lumbricoides*, indicating greater variation over time compared with hookworm and *T. trichiura*. A closer inspection of the temporal random effect associated with each year (**Figure 3**) shows that, although there is considerable inter-year variation, there is evidence of a general decline in prevalence odds over time for all three STH species, after adjusting for the spatial distribution of the survey data. Bayesian space-time models were also run on a restricted dataset comprising surveys that reported using Kato Katz for diagnosis of infection, with no major differences in findings observed (see **Text S2**).

For each STH species, and for the combined prevalence of STH, models consistently had an AUC (area under the ROC curve) estimate >0.85 for each prevalence threshold investigated, and in most instances AUC >0.9, indicating very good discriminatory performance of the model to classify areas (sensitivity and specificity) (**Table 3**) [36]. For comparison, at a 20% prevalence threshold the AUC was 0.91 (95% CI 0.89, 0.93) for the full space-time model, compared with 0.86 (0.81, 0.91) in a model that only accounted for spatial correlation, 0.80 (0.76, 0.83) when accounting for only temporal clustering, and 0.6 (0.56, 0.64) when neither of these random effects were included. Mean error of the space-time model predictions was <3% for each STH species, and 5% for combined prevalence, suggesting that the models consistently under-predicted prevalence.

Predictive prevalence and probability contour maps

Predictive maps of combined STH prevalence in school-aged children (i.e. school-based surveys using Kato-Katz for diagnosis) were created from the spatial model for two example years (1989 and 2009), shown in **Figure 4a–b.** Masked areas shown in grey have a mean LST >40°C, and so were considered outside the environmental limits for STH transmission. Clusters of high combined STH prevalence were located on the shores of Lake Victoria in western Kenya, and along the coast. Consistent with the temporal random effects shown in Figure 3, there are notable differences in predicted combined prevalence between years; overall prevalence was considerably lower in 2009, with predicted combined prevalence of 10 to 20% for much of the country, whilst in 1989 this was higher at >20–30%. Continuous probability contour maps are presented in **Figure 4c–d**. Areas within the red

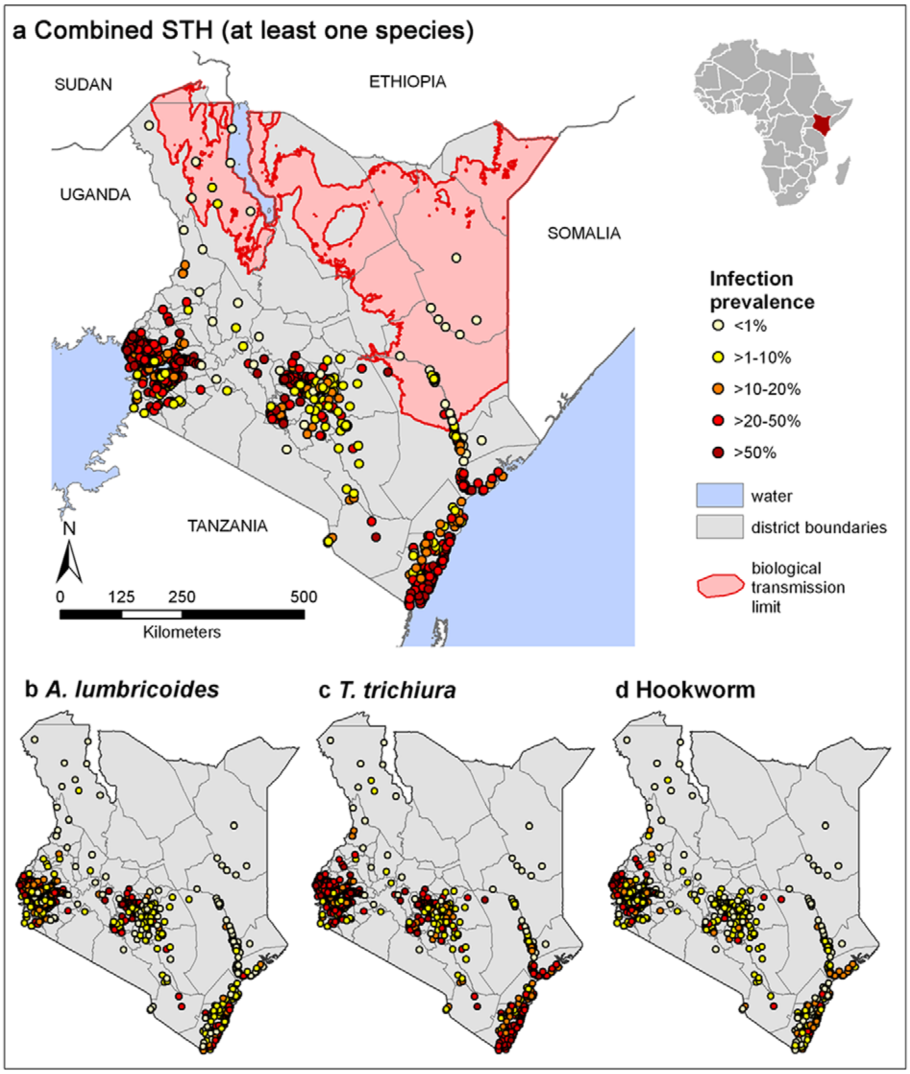

Figure 2. Distribution of soil-transmitted helminth survey data. Empirical prevalence of (**a**) combined soil-transmitted helminth, (**b**) *A. lumbricoides*, (**c**) *T. trichiura* and (**d**) hookworm infection from 945, cross-sectional surveys in Kenya 1974-2009. Combined prevalence was estimated calculated using a simple probabilistic model of combined infection, incorporating a small correction factor to allow for non-independence between species [20]. Biological transmission limits were estimated using maximum land surface temperature (LST), assuming no transmission when maximum LST exceeds 40{degree}C.
doi:10.1371/journal.pntd.0000958.g002

colour range (indicating probabilities of at least 70%) are those where there is a high probability that the mass drug administration (MDA) intervention threshold of 20% is exceeded, the blue areas (indicating probabilities of ≤ 30%) are those where there is a low probability that the 20% threshold is exceeded, whilst the yellow-orange areas (indicating probabilities of >30% but <70%) can be considered as areas of high uncertainty. The model for 2009 is characterised by generally low uncertainty, with pockets of higher uncertainty surrounding western and coastal regions (a consequence of the posterior mean prevalence being very close to 20%). In contrast, the 1989 model is characterised by high uncertainty for much of the country.

Predictive prevalence and probability contour maps for the three individual species are provided in **Text S2**. In brief, prevalence of hookworm infection was predicted to be low (<20%) throughout most of the country, with areas of higher prevalence in south-western Kenya (on the shores of Lake Victoria) and in Kilifi and Kwale districts on the coast. *A. lumbricoides* and *T. trichiura* infections had similar distributions, with high prevalence clusters in south-western Kenya and further north on the coast, in the districts of Lamu and Tana River. *A. lumbricoides* demonstrated greater variability over time, with an additional area of high infection prevalence identified in the Central Province in 1989.

Populations living in areas classified as endemic or hyperendemic in 2009

Intervention districts were defined as those where the population-weighted proportion of the district falling into a control-related endemicity class was greater than 33%, as shown in **Figure 5a**. Based on this modelling procedure, once yearly MDA with a benzamidazole, either albendazole or mebendazole, for all children of school-going age is recommended for 17 districts; Kilifi, Kwale, Lamu and Mombasa in Coast Province, Bondo, Gucha, Kisumu, Nyando, Nyamira and Siaya in Nyanza Province, Bureti, Kericho and Nandi South in the Rift Valley Province and Butere/Mumias, Mount Elgon, Bungoma and

Table 1. Summary of the Kenya soil-transmitted helminth survey data.

	Survey data points	Persons examined	Estimated combined STH prevalence (SD)
**Total**	945	106,370	37.0% (27.7%)
**Upper age sampled**			
<5 years	46	9915	39.1% (26.9%)
5–9 years	820	92239	36.4% (27.9%)
10–14 years	79	4216	44.6% (21.8%)
**Decade**			
1974–1979	39	10840	20.7% (20.0%)
1980–1989	205	34202	42.3% (22.9%)
1990–1999	183	20058	61.2% (27.5%)
2000–2009	513	41270	29.0% (24.2%)
**Province**			
Central	169	24178	34.3% (21.1%)
Coast	215	20576	41.3% (30.0%)
Eastern	77	15350	13.3% (9.9%)
Nairobi	2	250	76.2% (28.2%)
North eastern	18	1181	0.2% (0.6%)
Nyanza	371	36534	42.1% (23.0%)
Rift Valley	35	4034	12.2% (15.0%)
Western	58	4267	80.7% (21.8%)
**Type of sample survey**			
School	918	98750	38.2% (27.7%)
Community	27	7620	22.0% (20.0%)
**Method of diagnosis**			
Kato-Katz	557	66920	38.6% (30.4%)
All others*	388	39450	36.6% (23.3%)

*Other diagnostic methods include concentration (12 surveys), sedimentation (22 surveys), direct smear (12 surveys) and 342 studies for which the diagnostic method was not reported.

doi:10.1371/journal.pntd.0000958.t001

Kakamega in Western Province. Twice yearly MDA is recommended for Busia in Western Province, where 54% of the district's population are estimated to live in a hyperendemic area (prevalence ≥50%). These districts represent approximately 26% (2.82 million) of Kenyan children of school-going age. Thus, assuming a primary-school net enrolment rate of 81.5%, this

Table 2. Estimates of Bayesian hierarchical logistic regression models of soil-transmitted helminth species in Kenya. Data from 1974–2009.

	Posterior mean OR (95% CI)					
**Variable**	*A. lumbricoides*		**Hookworm**		*T. trichiura*	
Community based (vs. school based)	0.72	(0.51,0.96)	0.85	(0.57, 1.21)	0.45	(0.29, 0.68)
Other diagnostic method (vs. KK)	0.90	(0.72,1.1)	0.68	(0.53, 0.84)	0.43	(0.31, 0.57)
Maximum LST *	1.18	(0.93,1.46)	0.84	(0.73, 0.96)	0.83	(0.73, 0.99)
Elevation *	1.32	(0.84,1.80)	0.74	(0.60, 0.95)	0.75	(0.61, 0.93)
Precipitation *	1.01	(0.87,1.15)	1.13	(0.98, 1.28)	0.91	(0.77, 1.04)
EVI	2.57	(0.75,5.86)	0.62	(0.11, 1.64)	3.58	(0.47, 8.16)
Distance to permanent water bodies	0.77	(0.26,1.92)	1.24	(0.33, 3.14)	1.20	(0.34, 3.19)
ϕ (rate of decay of spatial correlation)	6.83	(4.94, 9.02)	8.62	(6.35, 10.93)	7.06	(5.15, 9.15)
σ^2_{space} (variance of spatial random effect)	4.03	(3.05,3.97)	3.53	(2.83,4.53)	3.55	(2.78, 4.63)
σ^2_{time} (variance of temporal random effect)	1.22	(0.56, 2.45)	0.66	(0.32, 1.36)	0.87	(0.39, 1.67)

CI, Bayesian credible interval; EVI, enhanced vegetation index; Maximum LST, maximum land surface temperature.
*Variables were standardised to have a mean of 0 and a standard deviation of 1.

doi:10.1371/journal.pntd.0000958.t002

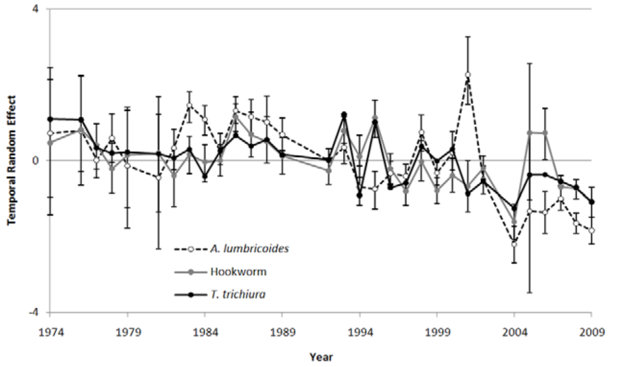

Figure 3. Temporal random effects for hookworm, *Ascaris lumbricoides* and *Trichuris trichiura* prevalence. Kenya from 1974–2009. Values are on a log scale (values of <0 indicate lower than average odds, values >0 indicate higher than average odds). Error bars show the 95% Bayesian Credible Intervals.
doi:10.1371/journal.pntd.0000958.g003

suggests that 2.36 million doses of a benzimidazole (albendazole or mebendazole) would be needed yearly if this model were to be used to guide decision makers as to where MDA should be offered.

For reference, **Figure 5b** shows the population-weighted proportion of each district exceeding the 20% prevalence threshold for 2009 and **Figure 5c** shows the projected population distribution in Kenya in 2009.

Discussion

Reliable and up-to-date maps of STH can help improve the geographical targetting and cost-effectiveness of school-based deworming. As global resources for deworming continue to increase there is an urgent need to strengthen our understanding of the spatial distribution of STH to inform planning at policy-relevant scales. Here, we present a new approach that combines GIS, geostatistics and high resolution environmental and population data, to map the predicted prevalence of STH in Kenya within a space-time Bayesian framework, incorporating data assembled from 945 independent, empirical helminth surveys spanning 34 years. The model distributions of combined STH prevalence had a high predictive accuracy (as demonstrated when splitting the database) and provide a description of how the observed STH prevalence has changed over time. This combined empirical and modelling approach providea a tool which could be used to guide decision making in helminth control in Kenya. The approach can also be extended to other endemic countries.

The 2009 risk map indicates that MDA is most warranted in western and coastal areas of the country, as indicated by a high probability that observed prevalence exceeds 20% (Figure 4c). Encouraging, an estimated 67% of the 2009 population live in areas where the predicted combined prevalence of STH is lower than 20%, including a large majority (57%) for whom we can be very certain (probability >0.7) that observed prevalence does not exceed 20%. This classification of endemicity differs from that predicted for 1989 and illustrates the importance of having up-to-date data to appropriately guide control. Our analysis indicates that observed combined STH prevalence has gradually decreased over time across the country, with populations experiencing lower observed prevalence of infection in more recent years. Consequently, several districts that in 1989 would have been indicated for targeted mass treatment based on the observed 20% prevalence threshold are not indicated for targeted mass treatment in 2009. Whilst the modelled extent of the temporal decline is subject to potential bias in measurement and selection, some of this decline may be attributable to small-scale implementation of deworming interventions, or may result from urbanisation and general improvements in socio-economic status, with associated improvements in living conditions, water and sanitation, and hygienic behaviour. For example, comparison of Demographic and Health Surveys from 1989 and 2009 reveals that access to an improved water source has risen nationally from 36.0% to 63.0% of households [37,38]. Such secular changes are probably not unique to Kenya and it is likely that much of Africa has experienced a gradual decline in the prevalence of parasitic diseases, including helminth infections. As deworming is scaled up across Africa and transmission continues to decline, it will become even more important to explicitly incorporate a time component into spatial models.

The models also serve to identify important determinants of observed infection patterns. The environmental and climatic associations presented here are consistent with the known biological determinants of helminth transmission [10,12]. Survey design (either community or school based) and diagnostic method are also important. In particular, the odds of hookworm and *T. trichiura* were significantly higher when surveys used the Kato-Katz method for diagnosis, confirming the increased sensitivity of this

Table 3. Measures of discriminatory ability, correlation, bias and accuracy of Bayesian hierarchical regression models.

Validation statistics	*A. lumbricoides*	Hookworm	*T. trichiura*	Combined STH
Area under the ROC curve (95% CI)				
5% threshold	0.92 (0.89, 0.93)	0.87 (0.83, 0.90)	0.91 (0.88, 0.94)	0.91 (0.886, 0.94)
10% threshold	0.92 (0.90, 0.94)	0.87 (0.84, 0.89)	0.90 (0.88, 0.92)	0.90 (0.87, 0.92)
20% threshold	0.92 (0.90, 0.94)	0.86 (0.84, 0.90)	0.91 (0.89, 0.94)	0.91 (0.89, 0.93)
50% threshold	0.87 (0.83, 0.90)	0.91 (0.90, 0.94)	0.92 (0.89, 0.96)	0.91 (0.89, 0.93)
Correlation coefficient	0.737	0.775	0.802	0.808
Mean error	−0.01	−0.01	−0.02	−0.03
Mean absolute error	0.09	0.11	0.08	0.14

ROC, receiver operating characteristic, a plot of sensitivity vs. one minus specificity; values of <0.7 indicate poor discriminatory performance, 0.7–0.8 acceptable, 0.8–0.9 excellent and >0.9 outstanding discriminatory performance. Correlation coefficient of 1 indicates perfect correlation between observed and expected prevalence. Mean error is the mean difference between observed and expected prevalence (assesses bias of estimates). Mean absolute error is the mean absolute difference between observed and expected prevalence (assesses accuracy of estimates).
doi:10.1371/journal.pntd.0000958.t003

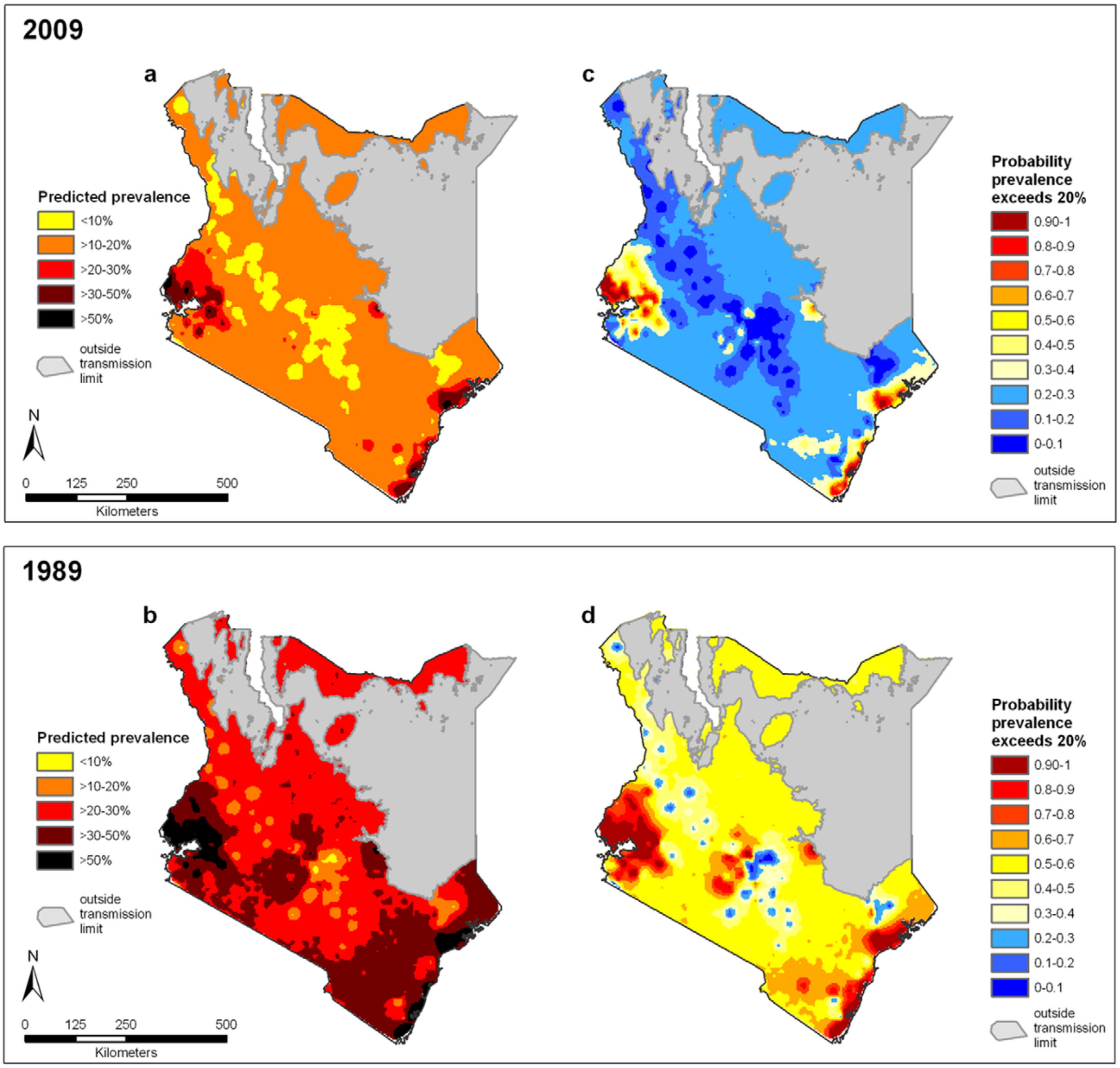

Figure 4. Continuous predicted combined soil-transmitted helminth (STH) prevalence and probability contour maps for Kenya. Probability contour map shows the probability that combined STH prevalence exceeds 20%. Estimates of predicted prevalence are the mean posterior predictive values from a Bayesian space-time model for (**a**) 2009 and (**b**) 1989. The probability contour maps show the spatial distribution of probability that combined STH prevalence is >20% for (**c**) 2009 and (**d**) 1989. Biological transmission limits were estimated using maximum land surface temperature (LST), assuming no transmission when maximum LST exceeds 40{degree}C.
doi:10.1371/journal.pntd.0000958.g004

method [39,40]. Our adopted Bayesian framework may in the future be expanded to include adjustment for measurement error of these different diagnostic tools. For example, recent work employing Bayesian methods has sought to estimate the sensitivity and specificity of diagnosis in the absence of a gold standard [31,41], and future refinements of our models will seek to reliable integrate measurement error into model predictions. Other sources of uncertainty also remain. For example, despite inclusion of environmental covariates, some small scale residual spatial variation remains - represented by the spatial random effects. This suggests that other factors, which may be related to poverty, hygiene and other small-scale environmental covariates, influence the observed current distribution of STH in Kenya. It should also be acknowledged that these maps represent stable rural populations; prevalence may vary among sub-populations such as nomads and refuges, or those living in urban slum areas with inadequate sanitation and overcrowding. In these instances, risk maps may under-estimate infection prevalence, and targeted surveys may be necessary before control decisions are made.

The explicit estimation of uncertainty is a particular strength of our adopted model-based geostatistics approach, which takes empirical estimates of infection prevalence and generates continuous maps by interpolating prevalence at unsurveyed locations on a grid system. Bayesian geostatistical inference acknowledges error or uncertainty associated with the data such that for each prediction location a distribution of possible prevalence values is generated (*i.e.* a posterior probability distribution). These uncertainties, which arise from the constraints of finite sampling, error in survey

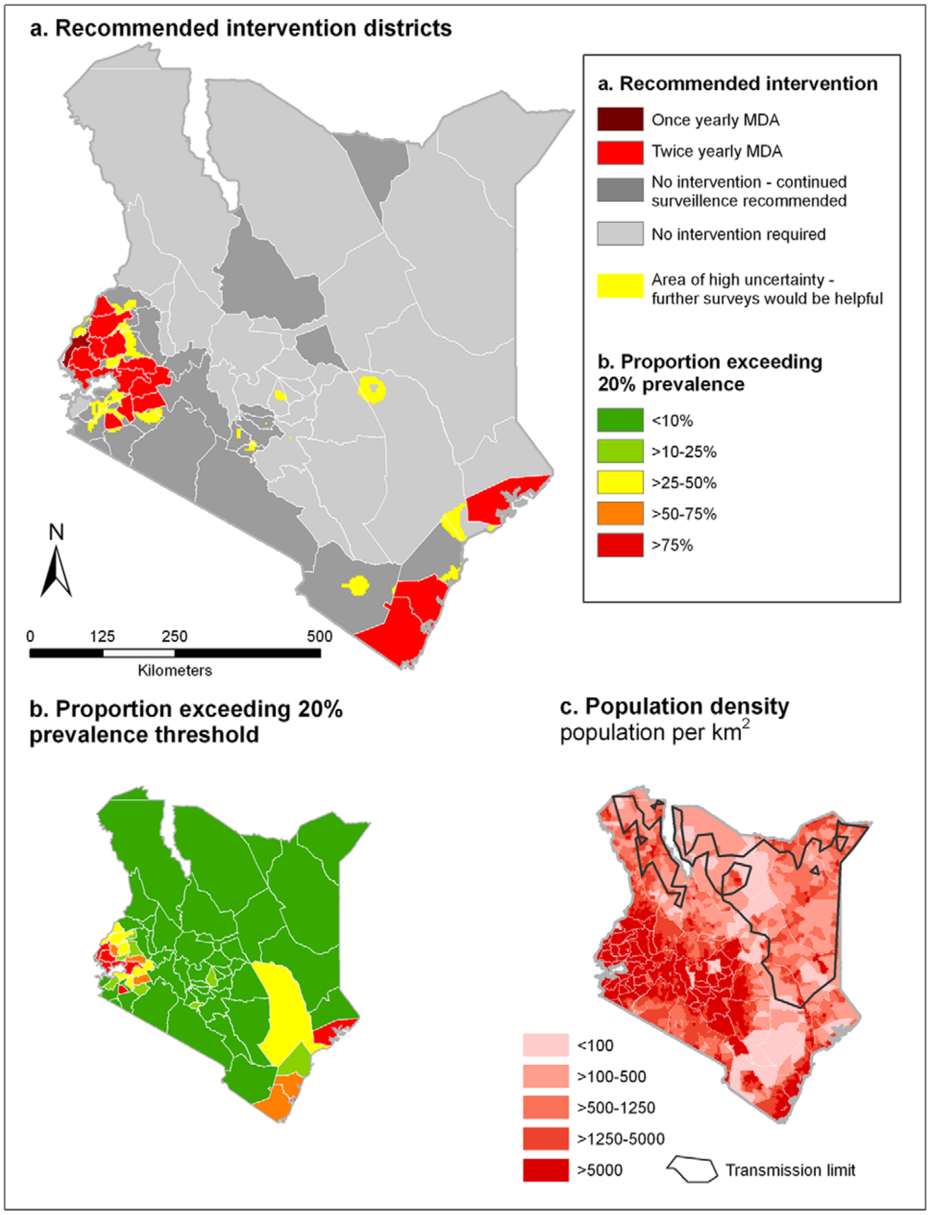

Figure 5. Control planning maps for Kenya in 2009. (**a**) Recommended intervention districts for 2009, and (**b**) the proportion of the population for each district exceeding the prevalence threshold. Prediction locations are defined as exceeding the prevalence threshold if the probability that prevalence is ≥20% is >0.5. Recommended intervention districts are defined as: once yearly mass drug administration (MDA), at least 33% of the district exceeds 20% prevalence threshold, and twice yearly MDA, at least 33% of the district exceeds 20% prevalence threshold. Continued surveillance is recommended for districts where historically >75% of the district exceeded the 20% prevalence based on 1998 and 1988, and areas of high uncertainty are those where we can only be 50–65% certain that prevalence is lower than 20%. For reference (**c**) shows the distribution of population density across Kenya, using the Gridded Population of Kenya (as previously described [34]), and the biological transmission limit, estimated using maximum LST >40°C.
doi:10.1371/journal.pntd.0000958.g005

measurements, uneven data distribution, and the inevitable presence of apparently random, unexplained, variation in prevalence, can have considerable implications for risk mapping approaches. For example, the WHO recommends implementation of targeted mass treatment for STH when the mean combined prevalence is ≥20% [19]. However, as a consequence of the degree of certainty, a predicted prevalence of 25% may or may not imply a high probability that the true prevalence exceeds 20% [16], as demonstrated in Figure 4b and 4d. An understating of these uncertainties can thus hugely assist decision making for disease control [42]. For example, the probability contour maps presented here help distinguish not only those areas where we can be certain that infection prevalence exceeds the 20% prevalence intervention threshold, but also those areas where we are still very uncertain and so where additional surveys are likely to be most helpful. For 2009, these areas of high uncertainty are few, and are limited to western and coastal areas close to recommended intervention districts.

An important limitation of traditional risk mapping strategies concerns spatial aggregation; that is, decision-making typically occurs at a district level, whereas environmental risk maps present continuous estimates. Surveys are often conducted in areas of known high endemicity, as reflected by the overall high prevalence

reported by the survey data. Mean prevalence may however vary significantly across districts, with localised pockets of high prevalence. In Kwale district for example, the predicted combined prevalence in 2009 varied between 5% and 53%, making the district-level mean prevalence of 21% harder to interpret. Risk maps are primarily restricted to continuous estimates due to the huge computational costs associated with prediction over large areas, constraining modelling approaches to those that produce independent, marginal predictions prediction that ignore correlation between prediction locations. Importantly, whilst these provide valid estimates of uncertainty for each location considered in isolation, it is not possible to construct valid probability distributions for a group of prediction locations, such as a district [43]. In the future, it may be feasible to develop joint simulation approaches that allow for robust estimates of mean prevalence at varying levels of spatial aggregation, such as those employed by Gething et al [43]. However, here we negate these problems by presenting the proportion of the population for each district that live in an area where prevalence exceeds the 20% threshold. This simple and statistically robust measurement provides an accurate indication of population at risk and represents a practical adaptation of modern Bayesian geostatistical techniques.

Bias associated with measurement error also represents an important limitation when interpreting the current model. The diagnostic sensitivity of different techniques can vary considerably [40,44,45], and whilst attempts were made to adjust for this, a large proportion of the surveys included did not mention the diagnostic method used nor the numbers of stool samples taken. Similarly, as the majority of surveys were school-based, selection bias may be present if the socio-demographic characteristics of school-going children varied through time or space. Notably, the proportion of surveys reporting using Kato Katz increased over time, whilst primary school enrolment rates have risen from 56% in 1998 to nearly 82% in 2009 [35]. The number of stool samples typically examined per individual may also have changed over time. Further, as infection intensities reduce as a consequence of the scale-up of control, the influence of measurement error in diagnosis will become more pronounced and must be taken into account. Specifically, a reduction in infection intensity would reduce test sensitivity, resulting in lower observed prevalence. Such systematic changes and potential biases may have served to dampen or exaggerate the temporal and spatial trends in modelled prevalence, and should be borne in mind during interpretation. However, restricting analysis to surveys that used Kato Katz did not lead to different conclusions. Current treatment guidelines are however based on prevalence as determined using the diagnostic tools currently available [16,32]. Thus, whilst the modelled distribution of STH infection may under-predict true prevalence, it does still provide a reliable indication of whether deworming is warranted given current WHO recommendations [16]. Further research is needed to investigate the full implications of diagnostic sensitivity and specificity for epidemiological surveillance and mapping of helminth species, particularly in the context of ongoing control.

A final limitation relates to how we estimate the combined prevalence of any STH species. In this instance, the distribution of each STH species was modelled independently and combined probability distributions were subsequently calculated post-estimation, using a probabilistic model that adjusts for non-independence between species [20]. Whilst this overcomes the considerable computational costs associated with joint modelling of all three species, it does risk over-estimating combined prevalence in areas of very low prevalence and conversely under-estimating prevalence in areas of very high prevalence. Despite this, we believe that this aspect is unlikely to have a major effect at a policy-decision level, which concerns mid-level prevalence values (20 to 50%).

Conclusion and future directions

Accurate and up-to-date maps of STH are essential for decision-making in helminth control. Our prediction of STH prevalence in 2009 highlights the marked heterogeneity of prevalence across Kenya and the importance of a geographically targeted approach to implementing control. The observed reduction in prevalence over time also serves to warn against relying on purely historic data when planning control; this will only become more important as control is implemented at increasing large scales. The resulting maps give an accurate description of the observed distribution of STH species across Kenya (together with associated uncertainties), and provide decision-makers with the information that can be used to prioritise districts for targeted STH control programmes among school-aged children, following the WHO recommendations.

The current work forms part of an ongoing project, the Global Atlas of Helminth Infection (www.thiswormyworld.org), which seeks to develop an open-access, global information resource on the distribution of STH and schistosomiasis [7,46]. The work presented here will be expanded to predict STH prevalence across all of sub-Saharan Africa. For each country, three types of maps will be presented: (i) a S*urvey Data Map* showing the observed prevalence of infection based on survey data; (ii) a *Predictive Risk Map* showing the probability that infection prevalence warrants MDA, according to recommended WHO thresholds; and (iii) a C*ontrol Planning Map* showing which districts require MDA treatment or where further surveys would be helpful in defining risk [47]. These maps will provide a sound epidemiological basis for guiding global investments in helminth control and country deworming efforts across the continent.

Supporting Information

Text S1 Model description.
Found at: doi:10.1371/journal.pntd.0000958.s001 (0.25 MB RTF)

Text S2 Additional results to support those provided in the main article.
Found at: doi:10.1371/journal.pntd.0000958.s002 (95.82 MB RTF)

Acknowledgments

We are grateful to the many individuals who contributed prevalence data to the Global Atlas of Helminth Infection, including Willis Akhwale, Mike Doenhoff, Thomas Handzel, Jimmy Kihara, Charles King, Tjalling Leenstra, Pascal Magnussen, Khalid Massa, Susan Montgomery, David Mwaniki, Mariam T Mwanje, Eric Muchiri, Annette Olsen, Evan Secor, Claire Standley and Russell Stothard. We are also grateful to Peris Karanja and Deborah Mbotha for their help with data assembly and Abdisalan Noor, Archie Clements and Ricardo Magalhães for statistical advice, and wish to thank Margaret Ndanyi, Karen Levy, Beth Waweru, Mariam T Mwanje, Jimmy Kihara, Lesley Drake, Don Bundy, Michael Kremer, Mike Mills and members of the Kenya school health steering committee for their contributions and support of deworming in Kenya. Thanks also go to Abdisalan Noor and to an anonymous reviewer for helpful comments on an earlier draft of the manuscript.

Author Contributions

Conceived and designed the experiments: SB. Performed the experiments: CWG. Analyzed the data: RLP PWG JLS HJWS. Wrote the paper: RLP CSM CWG SIH SB.

References

1. Warren KS, Bundy DAP, Anderson RM, Davis AR, Henderson DA, et al. (1993) Helminth infections. In: Jamison DT, Mosley WH, Measham AR, Bobadilla JL, eds. Disease Control Priorities in Developing Countries. Oxford: Oxford University Press. pp 131–160.
2. Hotez PJ, Bundy DAP, Beegle K, Brooker S, Drake L, et al. (2006) Helminth Infections: soil-transmitted helminth infections and schistosomiasis. In: Jamison DT, Breman JG, Measham AR, Alleyne G, Claeson M, et al., eds. Disease Control Priorities in Developing Countries. 2 ed. New York: Oxford University Press. pp 467–497.
3. Guyatt HL, Brooker S, Kihamia CM, Hall A, Bundy DA (2001) Evaluation of efficacy of school-based anthelmintic treatments against anaemia in children in the United Republic of Tanzania. Bull World Health Organ 79: 695–703.
4. Brooker S, Kabatereine NB, Fleming AF, Devlin N (2008) Cost and cost-effectiveness of nationwide school-based helminth control in Uganda: intra-country variation and effects of scaling-up. Health Policy Plan 23: 24–35.
5. Hopkins DR, Eigege A, Miri ES, Gontor I, Ogah G, et al. (2002) Lymphatic filariasis elimination and schistosomiasis control in combination with onchocerciasis control in Nigeria. Am J Trop Med Hyg 67: 266–272.
6. Ndyomugyenyi R, Kabatereine NB (2003) Integrated community-directed treatment for the control of onchocerciasis, schistosomiasis and intestinal helminths infections in Uganda: advantages and disadvantages. Trop Med Int Health 8: 997–1004.
7. Brooker S, Kabatereine NB, Smith JL, Mupfasoni D, Mwanje MT, et al. (2009) An updated atlas of human helminth infections: the example of East Africa. Int J Health Geogr 8: 42.
8. Brooker S, Michael E (2000) The potential of geographical information systems and remote sensing in the epidemiology and control of human helminth infections. Adv Parasitol 47: 245–288.
9. Brooker S, Clements ACA, Bundy DAP (2006) Global epidemiology, ecology and control of soil-transmitted helminth infections. Adv Parasitol 62: 221–261.
10. Clements AC, Deville M-A, Ndayishimiye O, Brooker S, Fenwick A (2010) Spatial co-distribution of neglected tropical diseases in the East African Great Lakes region: revisiting the justification for integrated control. Trop Med Int Health 15: 198–207.
11. Brooker S, Clements ACA (2009) Spatial heterogeneity of parasite co-infection: Determinants and geostatistical prediction at regional scales. International Journal for Parasitology 39: 591–597.
12. Raso G, Vounatsou P, Gosoniu L, Tanner HP, N'Goran E K, et al. (2006) Risk factors and spatial patterns of hookworm infection among schoolchildren in a rural area of western Cote d'Ivore. Int J Epidemiol 36: 201–210.
13. Clements AC, Bosque-Oliva E, Sacko M, Landoure A, Dembele R, et al. (2009) A comparative study of the spatial distribution of schistosomiasis in Mali in 1984-1989 and 2004-2006. PLoS Negl Trop Dis 3: e431.
14. Clements AC, Garba A, Sacko M, Toure S, Dembele R, et al. (2008) Mapping the probability of schistosomiasis and associated uncertainty, West Africa. Emerg Infect Dis 14: 1629–1632.
15. Vounatsou P, Raso G, Tanner M, N'goran EK, Utzinger J (2009) Bayesian geostatistical modelling for mapping schistosomiasis transmission. Parasitology 136: 1695–1705.
16. Diggle P, Thomson MC, Christensen OF, Rowlingson B, Obsomer V, et al. (2007) Spatial modelling and the prediction of *Loa loa* risk: decision making under uncertainty. Ann Trop Med Parasitol 101: 499–509.
17. Gyapong JO, Kyelem D, Kleinschmidt I, Agbo K, Ahouandogbo F, et al. (2002) The use of spatial analysis in mapping the distribution of bancroftian filariasis in four West African countries. Ann Trop Med Parasitol 96: 695–705.
18. Yang GL, Vounatsou P, Zhou X, Tanner M, Utzinger J (2005) A Bayesian-based approach for spatio-temporal modelling of county-level prevalence of *Schistosomiasis* infection in Jiangsu province, China. Int J Parasitol 131: 97–108.
19. WHO (2006) Preventive chemotherapy in human helminthiasis. Coordinated use of anthelmintihic drugs in control interventions a manual for health professionals and programme managers. Geneva: World Health Organization.
20. de Silva N, Hall A (2010) Using the prevalence of individual species of intestinal nematode worms to estimate the combined prevalence of any species. PLoS Negl Trop Dis 4: e655.
21. Noor AM, Gething PW, Alegana VA, Patil AP, Hay SI, et al. (2009) The risks of malaria infection in Kenya in 2009. BMC Infect Dis 9: 180.
22. WorldClim. http://www.worldclim.org/download.htm. Accessed 7th June 2009.
23. Hijmans RJ, Cameron SE, Parra JL, Jones PG, Jarvis A (2005) Very high resolution interpolated climate surfaces for global land areas. Int J Climatol 25.
24. Scharlemann JPW, Benz D, Hay SI, Purse BV, Tatem AJ, et al. (2008) Global data for ecology and epidemiology: a novel algorithm for temporal Fourier processing MODIS data. PLoS ONE 3: e1408.
25. Olson DM, Dinerstein E (2009) The Global 200: Priority ecoregions for global conservation. Ann Missouri Botanical Garden 89: 125–126.
26. Beer RJ (1976) The relationship between *Trichuris trichiura* (Linnaeus 1758) of man and *Trichuris suis* (Schrank 1788) of the pig. Res Vet Sci 20: 47–54.
27. Seamster AP (1950) Developmental studies concenring the eggs of *Ascaris lumbricoides* var. *suum.* Am Mid Natur 43: 450–468.
28. Udonsi JK, Atata G (1987) *Necator americanus*: Temperature, pH, Light, and Larval Development, Longevity, and Desiccation Tolerance. Exp Parasitol 63: 136–142.
29. Smith G, Schad GA (1989) *Ancylostoma duodenale* and *Necator americanus*: effect of temperature on egg development and mortality. Parasitol 99: 127–132.
30. Diggle P, Moyeed R, Tawn J (1998) Model-based geostatistics (with discussion). Appl Stats 47: 299–350.
31. Wang X-H, Zhou X-N, Vounatsou P, Chen Z, Utzinger J, et al. (2008) Bayesian spatio-temporal modelling of *Schistosoma japonicum* prevalence data in the absence of a diagnostic 'gold' standard. PLoS Negl Trop Dis 2: e250.
32. Montresor A, Crompton DWT, Hall A, Bundy DAP, Savioli L (1998) Guidelines for the evaluation of soil-transmitted helminthiasis and schistosomiasis at community level: a guide for managers of control programmes. Geneva: World Health Organization.
33. WHO (1994) Bench aids for the diagnosis of intestinal parasites. Geneva: World Health Organization.
34. Hay SI, Guerra CA, Gething PW, Patil AP, Tatem AJ, et al. (2009) A world malaria map: *Plasmodium falciparum* endemicity in 2007. PLoS Med 24: e1000048.
35. World Development Indicators (WDI) 2009 Washington D.C.: World Bank; 2009.
36. Brooker S, Hay SI, Bundy DAP (2000) Tools from ecology: useful for evaluating infection risk models. Trends Parasitol 18: 70 - 74.
37. Kenya National Bureau of Statistics, Nairobi, Kenya and MEASURE DHS, ICF Macro, Calverton, Maryland, USA (2009) Kenya: DHS, 2008-09 - Final Report.
38. National Council for Population and Development, Nairobi, Kenya and Institute for Resource Development/Macro Systems, Inc., Columbia, Maryland USA (1989) Kenya: DHS, 1989.
39. Goodman D, Haji HJ, Bickle QD, Stoltzfus RJ, Tielsch JM, et al. (2007) A comparison of methods for detecting the eggs of *Ascaris*, *Trichuris*, and hookworm in infant stool, and the epidemiology of infection in Zanzibari infants. Am J Trop Med Hyg 76: 725–731.
40. Knopp S, Mgeni AF, Khamis IS, Steinmann P, Stothard JR, et al. (2008) Diagnosis of soil-transmitted helminths in the era of preventive chemotherapy: effect of multiple stool sampling and use of different diagnostic techniques. PLoS Negl Trop Dis 2: e331.
41. Tarafder MR, Carabin H, Joseph L, Balolong EJ, Olveda R, et al. (2008) Estimating the sensitivity and specificity of Kato-Katz stool examination technique for detection of hookworms, *Ascaris lumbricoides* and *Trichuris trichiura* infections in humans in the absence of a 'gold standard'. Int J Parasitol 40: 399–404.
42. Clements AC, Lwambo NJ, Blair L, Nyandindi U, Kaatano G, et al. (2006) Bayesian spatial analysis and disease mapping: tools to enhance planning and implementation of a schistosomiasis control programme in Tanzania. Trop Med Int Health 11: 490–503.
43. Gething PW, Patil AP, Hay SI (2010) Quantifying aggregated uncertainty in *Plasmodium falciparum* malaria prevalence and populations at risk via efficient space-time geostatistical joint simulations. PloS Comp Biol 6: e1000724.
44. Goodman D, Haji HJ, Bickle QD, Stoltzfus RJ, Tielsch JM, et al. (2007) A comparison of methods for detecting the eggs of *Ascaris*, *Trichuris* and hookworm in infant stool, and the epidemiology of infection in Zanzibari infants. Am J Trop Med Hyg 76: 725–731.
45. Wang X-H, Zhou X-N, Vounatsou P, Chen Z, Utzinger J (2008) Bayesian Spatio-Temporal Modeling of Schistosoma japonicum Prevalence Data in the Absence of a Diagnostic 'Gold' Standard. PLoS Negl Trop Dis 2: e250.
46. Brooker S, Rowlands M, Haller L, Savioli L, Bundy DAP (2000) Towards an atlas of human helminth infection in sub-Saharan Africa: the use of geographical information systems (GIS). Parasitol Today 16: 303–307.
47. Brooker S, Hotez PJ, Bundy DAP (2010) The Global Atlas of Helminth Infection: Mapping the way forward in neglected tropical disease control. PLoS Negl Trop Dis 4(7): e779: e779.
