# Supplementary material for: The spatial-temporal distribution of soil-transmitted helminth infections in Guangdong Province, China: A geostatistical analysis of data derived from the three national parasitic surveys
Source: PLoS Negl Trop Dis. 2022 Jul 18;16(7):e0010622. doi: 10.1371/journal.pntd.0010622 (PMC9333454; doi:10.1371/journal.pntd.0010622)
Supplement: Attachment — Submitted filename: Response letter.docx [file pntd.0010622.s013.docx]

Guangzhou, 22^th^ May 2022

Response to Reviewer’s Comments

Dear editors and reviewers,

We refer to our manuscript titled “The spatial-temporal distribution of soil-transmitted helminth infections in Guangdong Province, China: a geostatistical analysis of data derived from the three national parasitic surveys” (your reference No. PNTD-D-22-00428). Many thanks to you and the reviewers for the excellent comments and suggestions. We have carefully read the comments and addressed all points proposed by reviewers in the revised manuscript. Our point-to-point response were provided below. **All line numbers in the response were referred to the clean version of the revised manuscript.**

***********************

Reviewer's Responses to Questions

Key Review Criteria Required for Acceptance?

As you describe the new analyses required for acceptance, please consider the following:

**Methods**

-Are the objectives of the study clearly articulated with a clear testable hypothesis stated?

-Is the study design appropriate to address the stated objectives?

-Is the population clearly described and appropriate for the hypothesis being tested?

-Is the sample size sufficient to ensure adequate power to address the hypothesis being tested?

-Were correct statistical analysis used to support conclusions?

-Are there concerns about ethical or regulatory requirements being met?

Reviewer #1: Overall, I think the methods are reasonable. The only major concern I have is that when compared with Ref 29, are there any improvements in the methods, or the methods used here are exactly the same as in Ref 29 and the only difference is the input data?

**Reply:**

Thanks to the reviewer for taking time to review our manuscript. Indeed, the methods of our study were similar to Ref 29, except that we accounted age and gender as fixed effects to construct the model, thus age- and gender-specific infection risk could be estimated. We added the related illustration in the ‘statistical analysis’ subsection (**lines 228-229 in the revised manuscript**).

Minor comments:

L80: "standardized prevalence rate" - how was it standardized?

**Reply:**

The standardized rates are rates adjusted to the same population structure, for reasonable comparison of rates in populations with different structures. Here, the standardized prevalence rate was the rate adjusted to the age and gender structures of the national population. We added the related illustration in the ‘introduction’ (**line 81 in the revised manuscript**).

L100: Why the infection rate (6.37%) is different from that presented on L98 (2.79%)?

**Reply:**

The detection rate of 2.79% presented on L98 of the original manuscript is the raw prevalence rate, calculated as the number of positive individuals divided by the number of examined ones. The rate of 6.37% in L100 of the original manuscript is the weighted infection rate, calculated using the sampling weights and population structure weights of sampled individuals. The weighted infection rate is more representative for the population rate than the raw detection rate. However, the first and the second national surveys did not report either the weighted infection rates or the standardized infection rate in Guangdong. To show a temporal trend here, we used the detection rate to describe the results from the three national surveys. To compare the current risk of infection in Guangdong with the national average level, we used the weighted infection rates, which was reported in the third national survey. Weadded related explanation of the two rates in the ‘introduction’ (**lines 97-98 and 102-103 in the revised manuscript**).

L105-108: Is this true for the whole China too?

**Reply:**

Indeed, researches on situation of STH infections in China were also mostly based on simply summarizing results from survey locations, hard to reflect the infection risk at high spatial resolution across the study region. Except that there was one study on high-resolution mapping of STH infections in China. However, this study based on observed data between 2000 and 2010, without considering age- and gender-specific infection risk, thus is not able to reflect both the long-term spatial-temporal trend and current status of infection risk in different genders and age groups. The description of this study is put in **lines 119-123 in the revised manuscript**.

L117: Since Ref 29 was also conducted by the same research group, I suspect that you also have access to the nationwide data? Why only map Guangdong data in the current study.

**Reply:**

Actually, we do not have access to the survey data of the whole China in the first and the third national surveys. Currently, we get access to the three national survey data in Guangdong Province from Guangdong Provincial Center for Disease Control and Prevention. Thus, the analysis is focus on Guangdong province.

L157: Could you be more specific about "according to the geographical location or natural environment" (i.e., east, middle, west? or plain and mountains?) and how many sectors there were?

**Reply:**

There were four sectors divided in each survey. However, as the methods for dividing the sectors in the three surveys were a little different, we revised the manuscript and described the methods separately for each survey, as following: “According to the geographical location, landscape or natural environment, the province was divided to four sectors. Particularly, in the first national survey, Guangdong Province was divided into sectors by the geographical location (i.e., east, west, north and south) and landscape (e.g., plain, hilly region, and mountainous region); in the second survey, the province was divided according to the geographical location; and in the third survey, the province was divided based on natural environment according to the *Ecozone Classification in China*.” (**lines 163-169 in the revised manuscript**). The more details were provided by the references **Ref 15**, **Ref 38**, and **Ref 39** listed in the revised manuscript.

L160: "thirdly, several sample units..." - How many sample units and how to determine the number of sample units for each county?

**Reply:**

We answered the above questions as following: “The total sampling size was calculated and distributed to the types of counties proportional to the population. The sampling unit was set as the natural village/community, each of which was sampled around 500 residents in the first and the second surveys and around 250 in the third one. The number of sampling units in each county type was calculated as the number of sampling size of the county type divided by the sample residents in each unit. Usually, each county sampled around 3 units and the number of sampled counties in each type were determined.” (**lines 172-178 in the revised manuscript**).

L161: "All samplings were randomized". - What do you mean here?

**Reply:**

We clarified the sampling randomization as following: “The counties were firstly sampled among each type, and then the units in the counties were sampled. In each sampled unit, residents were further sampled. The above sampling process was randomized.” (**lines 178-181 in the revised manuscript**).

L175: For the land cover types, what about Grassland, village, and bare land? Are they included in "unclassified"?

**Reply:**

The original land cover data contained 17 land cover types, including: 1) evergreen needleleaf forests; 2) evergreen broadleaf forests; 3) deciduous needleleaf forests; 4) deciduous broadleaf forests; 5) mixed forests; 6) closed shrublands; 7) open shrublands; 8) woody savannas; 9) savannas; 10) grasslands; 11) permanent wetlands; 12) croplands; 13) urban and built-up lands; 14) cropland/natural vegetation mosaics; 15) permanent snow and ice; 16) barren; and 17) water bodies. And there were areas with null data. We reclassified land cover data into the following six categories based on between-class similarities: 1) forest (including evergreen needleleaf forests, evergreen broadleaf forests, deciduous needleleaf forests, deciduous broadleaf forests, and mixed forests); 2) shrublands and grass (including closed shrublands, open shrublands, woody savannas, savannas, and grasslands); 3) wet areas (including permanent wetlands and water bodies); 4) croplands (including croplands, and cropland/natural vegetation mosaics); 5) urban (including urban and built-up lands); and 6) other types and unclassified (including permanent snow and ice, barren, and areas with null data). Thus, the grasslands were included in the “shrublands and grass” and the barren lands were included in the “other types and unclassified”. The village is not included in the original 17 types. We clarified the re-classification of land cover data in the ‘environmental, socioeconomic and demographic data’ subsection (**lines 191-199 in the revised manuscript**).

Table1: The footnotes could be included as a column in the table.

**Reply:**

Thanks for the reviewer’s suggestion on Table 1. We tried to include the footnotes as a new column in the table. However, there were too many words in the corresponding column and the width of the table was beyond the margin. Thus, we kept the original table.

L198: What do you mean by "all categorical variables are dumbed"?

**Reply:**

“All categorical variables are dumbed” means that we put each categorical variable as dummy variable in the later modelling analysis. We modified the description in the ‘statistical analysis’ subsection (**lines 225-226 in the revised manuscript**).

L206: "Bernoulli distribution" - I think you mean "binomial distribution"? The outcome of a Bernoulli distribution is binary.

**Reply:**

We thank the reviewer for pointing out this mistake. We revised the original “Bernoulli distribution” into “binomial distribution” in **line 230 of the revised manuscript**.

L209: "age groups k (k = 1, 2, 3, 4, 5)" - What are the age ranges for each group?

**Reply:**

We clarified the age range for each age group and revised the manuscript as following: “age groups *k* (*k* = 1, 2, 3, 4, 5, indicating the age group of <7 years old, 7-14 years old, 15-44 years old, 45-59 years old, ≥60 years old, respectively)” (**lines 233-235 in the revised manuscript)**.

L227: "less informative priors" - LESS compared with what? I think you mean weak priors or noninformative priors?

**Reply:**

We thank the reviewer for pointing out this. Yes, we meant weakly informative priors here. We have modified the relevant description in the ‘statistical analysis’ subsection (**line 254 in the revised manuscript**).

L233: Why not use continuous variables directly in the model?

**Reply:**

As some continuous variables may have non-linear correlations with the outcome variable, we converted continuous variables into categorical ones to capture the non-linear relationships. We selected the best functional form (i.e., continuous or categorical) of continuous variables through the variable selection procedure. We have added the relevant description in the ‘variable selection’ subsection (**lines 260-262 in the revised manuscript**).

L255: ps could also be written as 1 - (1-pA)*(1 - pr)*(1-ph), which is easier to understand?

**Reply:**

We thank the reviewer’s suggestion. We have changed the expression of the formula accordingly (**line 289 in the revised manuscript**).

Reviewer #2: (No Response).

Reviewer #3: The study’s methodology was well built-up.

The objectives of the study was clearly stated with testable hypothesis.

Statistical analysis was correctly applied to support hypotheses and conclusions.

The surveys of were approved by the ethics committee, and a written consent form was obtained after oral explanation of objectives, procedures, and potential risks.

**Reply:**

Thanks for the reviewer’s positive comment on our manuscript.

--------------------

**Results**

-Does the analysis presented match the analysis plan?

-Are the results clearly and completely presented?

-Are the figures (Tables, Images) of sufficient quality for clarity?

Reviewer #1: L265: Maybe remove the commas in the number of individuals since commas are used both in the numbers and between numbers, which is confusing, or you could just remove this sentence, since the numbers are presented in table 2.

**Reply:**

Thanks for the reviewer’s suggestion. We have removed the commas in the number of individuals (**line 302 in the revised manuscript**).

Figs S2-10: Putting the age groups as the panel title could be easier for the readers to get the differences between panels

**Reply:**

We have put the age groups as panel titles in **Figs S2-10 in the supplementary information**.

S11 Text: Are these results based on the training or validating samples?

**Reply:**

The results of model validation in S11 Text based on both the training and validating sets. For model validation, we randomly sampled 80% of the observed locations as the training set, and the remaining 20% as the validating set. The training set was applied to fit the model, from which parameter estimates were obtained and were further used to estimate the age- and gender specific prevalence ($\hat{\pi}_{igk}$) at locations of the validating set. We compared the observed ($\pi_{igk}$) and estimated prevalence ($\hat{\pi}_{igk}$) at each observation of the validation set (with the total number of observations *N*), by calculating the mean error ($ME=\frac{1}{N}\times\sum_{i=1} \sum_{g=1} \sum_{k=1}(\pi_{igk}-\hat{\pi}_{igk})$), the mean absolute error ($MAE=\frac{1}{N}\times\sum_{i=1} \sum_{g=1} \sum_{k=1}｜\pi_{igk}-\hat{\pi}_{igk}｜$), the coverage of observations within 95% Bayesian credible interval (BCI) of posterior distribution of estimated prevalence, and the area under the receiver operating characteristic (ROC) curve (AUC) to evaluate the performance of the model. We have changed the relevant descriptions in the ‘model validation’ subsection (**lines 273-282 in the revised manuscript**).

Reviewer #2: (No Response)

Reviewer #3: The result presented clearly following the intended plan.

The risk maps appropriately denote the regions with higher STH prevalence with calculated standard deviation.

**Reply:**

Thanks for the reviewer’s positive comment on the results.

--------------------

**Conclusions**

-Are the conclusions supported by the data presented?

-Are the limitations of analysis clearly described?

-Do the authors discuss how these data can be helpful to advance our understanding of the topic under study?

-Is public health relevance addressed?

Reviewer #1: (No Response)

Reviewer #2: (No Response)

Reviewer #3: (No Response)

--------------------

**Editorial and Data Presentation Modifications?**

Use this section for editorial suggestions as well as relatively minor modifications of existing data that would enhance clarity. If the only modifications needed are minor and/or editorial, you may wish to recommend “Minor Revision” or “Accept”.

Reviewer #1: (No Response)

Reviewer #2: (No Response)

Reviewer #3: Please modify the minor points in your manuscript as mentioned below.

Lines 105 – ‘spatial targeted’ to ‘spatially targeted’ or ‘spatial-targeted.’

Lines 228 – It might be better to explain why the authors applied less informative prior distributions for Bayesian models.

Lines 398 – ‘uses’ to ‘used.’

**Reply:**

Thanks for the reviewer’s suggestions. We have modified the above two typing errors accordingly (**lines 108 and 444 in the revised manuscript**). Moreover, as we did not have enough prior information on the distributions of the parameters in the Bayesian models, weakly informative prior distributions were adopted, to avoid subjectively selecting unreasonable informative priors. We added the above explanation in the ‘statistical analysis’ subsection (**lines 252-257 in the revised manuscript**).

--------------------

**Summary and General Comments**

Use this section to provide overall comments, discuss strengths/weaknesses of the study, novelty, significance, general execution and scholarship. You may also include additional comments for the author, including concerns about dual publication, research ethics, or publication ethics. If requesting major revision, please articulate the new experiments that are needed.

Reviewer #1: This manuscript use environmental and socio-economic factors to interpolate the point STHs prevalence survey data in Guangzhou to a raster layer. The methods and results are well-presented and easy to follow. Although the results might be valuable for understanding the spatial distribution and its changes across time in Guangdong, there is not much novelty in the methods if it is exactly the same as in Ref 29.

**Reply:**

We totally agreed that the methodology used in this manuscript is not much novel, as it is similar to that used in Ref 29. The aim of this study is to estimate the long-term spatial-temporal, age- and gender-specific infection risk of STHs in Guangdong Province, China, at high-spatial resolutions, thus, to provide important information assisting the control and prevention strategies. We used reasonable, instead of novel methods, to achieve the aim.

Reviewer #2: (No Response)

Reviewer #3: This original article is about “The spatial-temporal distribution of soil-transmitted helminth infections in Guangdong Province, China: a geostatistical analysis of data derived from the three national parasitic surveys.” The paper is well written in describing the distribution of STH infections in Guangdong Province. The study’s methodology was well built-up, and the map drawn by the survey seems to play an essential role in implementing control programs.

There are some suggestions for authors,

1. If the survey data are not available, sharing R-codes with the public by data repositories like GitHub will be a good alternative.

2. How about presenting the ‘probability prevalence exceeds threshold map’? The threshold can impose the overall prevalence of China or the Province, indicating the regions require control programs in priority.

**Reply:**

We thank the reviewer for the wonderful suggestions. We have shared R-codes used in this study with the public in GitHub (<https://github.com/SYSU-STH/Spatial-temporal-mapping-of-STH>). We added the related illustration in ‘high-resolution maps’ subsection (**line 297 in the revised manuscript**).

Besides, we also produced maps of the probability that prevalence exceeds a certain threshold for any STH infection. The prevalence thresholds for preventive chemotherapy (20%) and transmission control (1%) were used (**lines 291-294 in the revised manuscript in the Methods part**). The related results were added in the ‘estimated risk maps’ subsection (**lines 376-378, 379, and 382-384, and Fig 6 in the revised manuscript**).

--------------------

**References**

We added some new references, and the order of the references has been modified accordingly.

Besides, after carefully checking the whole manuscript, we found several typing errors in the results, which were corrected in the revised manuscript.
